
JOURNAL INFORMATION
==============================
NLM Title Abbreviation: J Int AIDS Soc
Journal ID: J Int AIDS Soc
NLM Journal ID: 101478566
Journal ID: JIAS

Journal of the International AIDS Society

EISSN: 1758-2652
Publisher: Wiley

ARTICLE INFORMATION
==============================
PMCID: PMC3499900
DOI: 10.7448/IAS.15.5.18439
Article ID: 18439
Article version: 1
Subjects: AIDS2012 Abstract Supplement

Track B Clinical Science

Electronic publication date: 2012 Oct 22
Publication date: 2012
Volume: 15
Issue: Suppl 3
Electronic Location ID: 18439
Copyright: © 2012 Track B Clinical Science.
Copyright year: 2012
License: This is an open-access article distributed under the terms of the Creative Commons Attribution License, which permits unrestricted use, distribution, and reproduction in any medium, provided the original work is properly cited.
License URL: https://creativecommons.org/licenses/by/2.0/

==============================
JOURNAL INFORMATION
==============================
NLM Title Abbreviation: J Int AIDS Soc
Journal ID: J Int AIDS Soc
NLM Journal ID: 101478566
Journal ID: JIAS

Journal of the International AIDS Society

EISSN: 1758-2652
Publisher: Wiley

ARTICLE INFORMATION
==============================
Article ID: 18439-01
Subjects: B1 - Impact of co-factors: viral clade, tropism, genetic factors, age and gender on disease progression

MOAB0104: Toll-like receptor 4 polymorphism influence the development of opportunistic diseases in HIV-positive patients

Kyrychenko T. 1 §
Dubynska G. 2
Koval T. 2
Kaidashev I. 2
Korshenko V. 1
1 Poltava Regional HIV/AIDS Prevention and Control Center, Poltava, Ukraine
2 Ukrainian Medical and Dental Academy, Poltava, Ukraine
§ Presenting author email: loyal7@rambler.ru
Electronic publication date: 2012 Oct 22
Publication date: 2012
Volume: 15
Issue: Suppl 3
Electronic Location ID: 18439-01
Copyright: © 2012 T. Kyrychenko et al.
Copyright year: 2012
License: This is an open-access article distributed under the terms of the Creative Commons Attribution License, which permits unrestricted use, distribution, and reproduction in any medium, provided the original work is properly cited.
License URL: http://creativecommons.org/licenses/by/2.0/

JOURNAL INFORMATION
==============================
NLM Title Abbreviation: J Int AIDS Soc
Journal ID: J Int AIDS Soc
NLM Journal ID: 101478566
Journal ID: JIAS

Journal of the International AIDS Society

EISSN: 1758-2652
Publisher: Wiley

ARTICLE INFORMATION
==============================
Article ID: 18439-02
Subjects: B2 - Acute and early infection

TUPDB0202: Prospective detection and description of acute HIV infection in a high risk cohort of women in resource-limited settings: identifying factors that contribute to the continued spread of HIV

Rono K. 1 §
Kibuuka H. 2
Maganga L. 3
Kosgei J. 1
Sekiziyivu A. 2
Sanga E. 3
Ngetich E. 1
Bolen Valenzuela A. 4
Michael N. 4
Robb M. 4
1 Kenya Medical Research Institute-Walter Reed Project, Kericho, Kenya
2 Makerere University Walter Reed Project, Kampala, Uganda
3 Mbeya Medical Research Programme, Mbeya, United Republic of Tanzania
4 US Military HIV Research Program, Bethesda, United States
§ Presenting author email: krono@wrp-kch.org
Electronic publication date: 2012 Oct 22
Publication date: 2012
Volume: 15
Issue: Suppl 3
Electronic Location ID: 18439-02
Copyright: © 2012 K. Rono et al.
Copyright year: 2012
License: This is an open-access article distributed under the terms of the Creative Commons Attribution License, which permits unrestricted use, distribution, and reproduction in any medium, provided the original work is properly cited.
License URL: http://creativecommons.org/licenses/by/2.0/

JOURNAL INFORMATION
==============================
NLM Title Abbreviation: J Int AIDS Soc
Journal ID: J Int AIDS Soc
NLM Journal ID: 101478566
Journal ID: JIAS

Journal of the International AIDS Society

EISSN: 1758-2652
Publisher: Wiley

ARTICLE INFORMATION
==============================
Article ID: 18439-03
Subjects: B2 - Acute and early infection

TUPDB0204: Very early initiation of combination antiviral therapy results in normal levels of markers of immune activation

Markowitz M. 1 §
Evering T. 1
Figueroa A. 1
Rodriguez K. 1
La Mar M. 1
Garmon D. 1
Sahi V. 1
Mohri H. 1
1 Aaron Diamond AIDS Research Center, New York, United States
§ Presenting author email: mmarkowitz@adarc.org
Electronic publication date: 2012 Oct 22
Publication date: 2012
Volume: 15
Issue: Suppl 3
Electronic Location ID: 18439-03
Copyright: © 2012 M. Markowitz et al.
Copyright year: 2012
License: This is an open-access article distributed under the terms of the Creative Commons Attribution License, which permits unrestricted use, distribution, and reproduction in any medium, provided the original work is properly cited.
License URL: http://creativecommons.org/licenses/by/2.0/

JOURNAL INFORMATION
==============================
NLM Title Abbreviation: J Int AIDS Soc
Journal ID: J Int AIDS Soc
NLM Journal ID: 101478566
Journal ID: JIAS

Journal of the International AIDS Society

EISSN: 1758-2652
Publisher: Wiley

ARTICLE INFORMATION
==============================
Article ID: 18439-04
Subjects: B3 - Elite and viremic controllers

WEAX0105: HLAB57 does not fully explain the ability of HIV controllers to spontaneously clear hepatitis C virus (HCV) infection

Asher A. 1 2 §
Santos G.-M. 1
Dokubo E.K. 3
Martin J. 4
Deeks S. 4
Tobler L. 5
Busch M. 5
Hunt P. 4
Page K. 1
1 University of California San Francisco, Epidemiology & Biostatistics, San Francisco, United States
2 University of California San Francisco School of Nursing, Community Health Systems, San Francisco, United States
3 University of California San Francisco, Center for AIDS Prevention Studies, San Francisco, United States
4 University of California San Francisco, Positive Health Program, San Francisco, United States
5 Blood Systems Research Institute, San Francisco, United States
§ Presenting author email: alice.asher@ucsf.edu
Electronic publication date: 2012 Oct 22
Publication date: 2012
Volume: 15
Issue: Suppl 3
Electronic Location ID: 18439-04
Copyright: © 2012 A. Asher et al.
Copyright year: 2012
License: This is an open-access article distributed under the terms of the Creative Commons Attribution License, which permits unrestricted use, distribution, and reproduction in any medium, provided the original work is properly cited.
License URL: http://creativecommons.org/licenses/by/2.0/

JOURNAL INFORMATION
==============================
NLM Title Abbreviation: J Int AIDS Soc
Journal ID: J Int AIDS Soc
NLM Journal ID: 101478566
Journal ID: JIAS

Journal of the International AIDS Society

EISSN: 1758-2652
Publisher: Wiley

ARTICLE INFORMATION
==============================
Article ID: 18439-05
Subjects: B5 - Disease burden - morbidity/mortality

THAB0304: Trends over time in underlying causes of death in the D:A:D study from 1999 to 2011

Weber R. 1
Smith C. 2 §
D:A:D Study Group
1 University Hospital, Zurich, Switzerland
2 UCL, Infection and Population Health, London, United Kingdom
§ Presenting author email: c.smith@ucl.ac.uk
Electronic publication date: 2012 Oct 22
Publication date: 2012
Volume: 15
Issue: Suppl 3
Electronic Location ID: 18439-05
Copyright: © 2012 C. Smith et al.
Copyright year: 2012
License: This is an open-access article distributed under the terms of the Creative Commons Attribution License, which permits unrestricted use, distribution, and reproduction in any medium, provided the original work is properly cited.
License URL: http://creativecommons.org/licenses/by/2.0/

JOURNAL INFORMATION
==============================
NLM Title Abbreviation: J Int AIDS Soc
Journal ID: J Int AIDS Soc
NLM Journal ID: 101478566
Journal ID: JIAS

Journal of the International AIDS Society

EISSN: 1758-2652
Publisher: Wiley

ARTICLE INFORMATION
==============================
Article ID: 18439-06
Subjects: B7 - HIV testing, including new algorithms and strategies

THAB0301: Non-adherence to HIV testing guidelines and late HIV diagnosis is common among U.S. black men who have sex with men (MSM)

Mannheimer S. 1 2 §
Wang L. 3
Tieu H.V. 4
del Rio C. 5
Buchbinder S. 6
Wilton L. 7
Glick S. 8
Cummings V. 9
Mayer K.H. 10
on behalf of the HPTN 061 Study Team
1 Harlem Hospital/ Columbia University, Medicine, New York, United States
2 Mailman School of Public Health, Columbia University, Epidemiology, New York, United States
3 Statistical Center for HIV/AIDS Research & Prevention (SCHARP), Seattle, United States
4 New York Blood Center, New York, United States
5 Emory University Rollins School of Public Health, Department of Global Health, Atlanta, United States
6 San Francisco Department of Health, HIV Research Section, San Francisco, United States
7 Binghamton University, Department of Human Development, Binghamton, United States
8 The George Washington University School of Public Health and Health Services, Department of Epidemiology and Biostatistics, Washington, United States
9 Johns Hopkins University Medical School, Pathology Department, Baltimore, United States
10 The Fenway Institute/Harvard Medical School, Boston, United States
§ Presenting author email: sbm20@columbia.edu
Electronic publication date: 2012 Oct 22
Publication date: 2012
Volume: 15
Issue: Suppl 3
Electronic Location ID: 18439-06
Copyright: © 2012 S. Mannheimer et al.
Copyright year: 2012
License: This is an open-access article distributed under the terms of the Creative Commons Attribution License, which permits unrestricted use, distribution, and reproduction in any medium, provided the original work is properly cited.
License URL: http://creativecommons.org/licenses/by/2.0/

JOURNAL INFORMATION
==============================
NLM Title Abbreviation: J Int AIDS Soc
Journal ID: J Int AIDS Soc
NLM Journal ID: 101478566
Journal ID: JIAS

Journal of the International AIDS Society

EISSN: 1758-2652
Publisher: Wiley

ARTICLE INFORMATION
==============================
Article ID: 18439-07
Subjects: B7 - HIV testing, including new algorithms and strategies

THAB0302: Cost-effectiveness of more frequent HIV screening of men who have sex with men in the United States

Hutchinson A. 1 §
Sansom S. 1
Farnham P. 1
1 Centers for Disease Control and Prevention, Division of HIV/AIDS Prevention, Atlanta, United States
§ Presenting author email: ash2@cdc.gov
Electronic publication date: 2012 Oct 22
Publication date: 2012
Volume: 15
Issue: Suppl 3
Electronic Location ID: 18439-07
Copyright: © 2012 A. Hutchinson et al.
Copyright year: 2012
License: This is an open-access article distributed under the terms of the Creative Commons Attribution License, which permits unrestricted use, distribution, and reproduction in any medium, provided the original work is properly cited.
License URL: http://creativecommons.org/licenses/by/2.0/

JOURNAL INFORMATION
==============================
NLM Title Abbreviation: J Int AIDS Soc
Journal ID: J Int AIDS Soc
NLM Journal ID: 101478566
Journal ID: JIAS

Journal of the International AIDS Society

EISSN: 1758-2652
Publisher: Wiley

ARTICLE INFORMATION
==============================
Article ID: 18439-08
Subjects: B7 - HIV testing, including new algorithms and strategies

TUPDB0203: Optimizing a dried blood spot-based pooled RT-PCR technique for identification of acute HIV infections in Mochudi, Botswana

Davis R. 1 §
Dzoro S. 1
Moyo S. 1
Gaseitsiwe S. 1 2
Musonda R. 1 2
Novitsky V. 1 2
Essex M. 1 2
1 Botswana-Harvard AIDS Institute Partnership, Gaborone, Botswana
2 Harvard School of Public Health AIDS Initiative, Immunology and Infectious Diseases, Boston, United States
§ Presenting author email: rdavis@jhsph.edu
Electronic publication date: 2012 Oct 22
Publication date: 2012
Volume: 15
Issue: Suppl 3
Electronic Location ID: 18439-08
Copyright: © 2012 R. Davis et al.
Copyright year: 2012
License: This is an open-access article distributed under the terms of the Creative Commons Attribution License, which permits unrestricted use, distribution, and reproduction in any medium, provided the original work is properly cited.
License URL: http://creativecommons.org/licenses/by/2.0/

JOURNAL INFORMATION
==============================
NLM Title Abbreviation: J Int AIDS Soc
Journal ID: J Int AIDS Soc
NLM Journal ID: 101478566
Journal ID: JIAS

Journal of the International AIDS Society

EISSN: 1758-2652
Publisher: Wiley

ARTICLE INFORMATION
==============================
Article ID: 18439-09
Subjects: B8 - Viral load testing, including point of care diagnostics

WEPDB0103: A field evaluation of HIV-1 RNA viral load in plasma and dried blood spots using the COBAS Amplicor Analyzer and the Nucleic Acid Extraction COBAS Ampliprep/Taqman in Kisumu, Kenya

Ouma K. 1 §
Basavaraju S. 2
Okonji J. 3
Williamson J. 4
Mills L. 4
Zeh C. 4
1 KEMRI-CDC Research and Public Health Collaboration, Kisumu, Kenya
2 Centers for Disease Control and Prevention, HIV Prevention Branch, Division of Global HIV/AIDS, Center for Global Health, Georgia, United States
3 KEMRI-CDC Research and Public Health Collaboration, HIV Research Laboratory, Kisumu, Kenya
4 Centers for Disease Control and Prevention, Division of HIV/AIDS Prevention, Kisumu, Kenya
§ Presenting author email: kouma@kemricdc.org
Electronic publication date: 2012 Oct 22
Publication date: 2012
Volume: 15
Issue: Suppl 3
Electronic Location ID: 18439-09
Copyright: © 2012 K. Ouma et al.
Copyright year: 2012
License: This is an open-access article distributed under the terms of the Creative Commons Attribution License, which permits unrestricted use, distribution, and reproduction in any medium, provided the original work is properly cited.
License URL: http://creativecommons.org/licenses/by/2.0/

JOURNAL INFORMATION
==============================
NLM Title Abbreviation: J Int AIDS Soc
Journal ID: J Int AIDS Soc
NLM Journal ID: 101478566
Journal ID: JIAS

Journal of the International AIDS Society

EISSN: 1758-2652
Publisher: Wiley

ARTICLE INFORMATION
==============================
Article ID: 18439-10
Subjects: B8 - Viral load testing, including point of care diagnostics

THPDB0102: Evaluation of the WHO immunologic criteria for antiretroviral treatment failure among adults on first line highly active antiretroviral therapy (HAART) in south India

Vallabhaneni S. 1 §
Chandy S. 2
Heylen E. 1
Ekstrand M.L. 1 2
1 University of California at San Francisco, Center for AIDS Prevention Studies, San Francisco, United States
2 St John's National Academy of Health Sciences, Medicine, Bangalore, India
§ Presenting author email: snigdhav@gmail.com
Electronic publication date: 2012 Oct 22
Publication date: 2012
Volume: 15
Issue: Suppl 3
Electronic Location ID: 18439-10
Copyright: © 2012 S. Vallabhaneni et al.
Copyright year: 2012
License: This is an open-access article distributed under the terms of the Creative Commons Attribution License, which permits unrestricted use, distribution, and reproduction in any medium, provided the original work is properly cited.
License URL: http://creativecommons.org/licenses/by/2.0/

JOURNAL INFORMATION
==============================
NLM Title Abbreviation: J Int AIDS Soc
Journal ID: J Int AIDS Soc
NLM Journal ID: 101478566
Journal ID: JIAS

Journal of the International AIDS Society

EISSN: 1758-2652
Publisher: Wiley

ARTICLE INFORMATION
==============================
Article ID: 18439-11
Subjects: B9 - Drug resistance testing

TUAB0304: High levels of drug resistance after failure of first-line antiretroviral therapy in rural South Africa: impact on standardised second-line regimens

Manasa J. 1 §
McGrath N. 1 2
Lessells R. 1 3
Skingsley A. 1
Newell M.-L. 1
de Oliveira T. 1
1 University of KwaZulu-Natal, Africa Centre for Health and Population Studies, Somkhele, South Africa
2 London School of Hygiene and Tropical Medicine, Infectious Disease Epidemiology, London,, United Kingdom
3 London School of Hygiene and Tropical Medicine, Clinical Research, London, United Kingdom
§ Presenting author email: jmanasa@gmail.com
Electronic publication date: 2012 Oct 22
Publication date: 2012
Volume: 15
Issue: Suppl 3
Electronic Location ID: 18439-11
Copyright: © 2012 J. Manasa et al.
Copyright year: 2012
License: This is an open-access article distributed under the terms of the Creative Commons Attribution License, which permits unrestricted use, distribution, and reproduction in any medium, provided the original work is properly cited.
License URL: http://creativecommons.org/licenses/by/2.0/

JOURNAL INFORMATION
==============================
NLM Title Abbreviation: J Int AIDS Soc
Journal ID: J Int AIDS Soc
NLM Journal ID: 101478566
Journal ID: JIAS

Journal of the International AIDS Society

EISSN: 1758-2652
Publisher: Wiley

ARTICLE INFORMATION
==============================
Article ID: 18439-12
Subjects: B9 - Drug resistance testing

THPDB0101: Accumulation of protease mutations among patients on non-suppressive 2nd-line ART in Nigeria

Rawizza H. 1 §
Chaplin B. 2
Meloni S. 2
Okonkwo P. 3
Kanki P. 2
the APIN PEPFAR Team
1 Brigham & Women's Hospital, Infectious Disease, Boston, United States
2 Harvard School of Public Health, Immunology and Infectious Disease, Boston, United States
3 AIDS Prevention Initiative In Nigeria (APIN), Abuja, Nigeria
§ Presenting author email: hrawizza@hsph.harvard.edu
Electronic publication date: 2012 Oct 22
Publication date: 2012
Volume: 15
Issue: Suppl 3
Electronic Location ID: 18439-12
Copyright: © 2012 H. Rawizza et al.
Copyright year: 2012
License: This is an open-access article distributed under the terms of the Creative Commons Attribution License, which permits unrestricted use, distribution, and reproduction in any medium, provided the original work is properly cited.
License URL: http://creativecommons.org/licenses/by/2.0/

JOURNAL INFORMATION
==============================
NLM Title Abbreviation: J Int AIDS Soc
Journal ID: J Int AIDS Soc
NLM Journal ID: 101478566
Journal ID: JIAS

Journal of the International AIDS Society

EISSN: 1758-2652
Publisher: Wiley

ARTICLE INFORMATION
==============================
Article ID: 18439-13
Subjects: B11 - CD4 testing, including point of care diagnostics

WEPDB0101: Frequent CD4 cell count monitoring is not necessary for persons with counts ≥350 cells/µl and viral suppression

Gale H. 1 §
Gitterman S. 1
Gordin F. 1 2
Benator D. 1 2
Kan V. 1 2
1 Veterans Affairs Medical Center, Infectious Diseases Section, Washington, United States
2 George Washington University Medical Center, Division of Infectious Diseases, Washington, United States
§ Presenting author email: howard.gale@va.gov
Electronic publication date: 2012 Oct 22
Publication date: 2012
Volume: 15
Issue: Suppl 3
Electronic Location ID: 18439-13
Copyright: © 2012 H. Gale et al.
Copyright year: 2012
License: This is an open-access article distributed under the terms of the Creative Commons Attribution License, which permits unrestricted use, distribution, and reproduction in any medium, provided the original work is properly cited.
License URL: http://creativecommons.org/licenses/by/2.0/

JOURNAL INFORMATION
==============================
NLM Title Abbreviation: J Int AIDS Soc
Journal ID: J Int AIDS Soc
NLM Journal ID: 101478566
Journal ID: JIAS

Journal of the International AIDS Society

EISSN: 1758-2652
Publisher: Wiley

ARTICLE INFORMATION
==============================
Article ID: 18439-14
Subjects: B12 - Opportunistic infections (excluding tuberculosis)

MOAB0102: Cost benefit of integrating cryptococcal antigen screening and preemptive treatment into routine HIV care

Meya D. 1 2
Rajasingham R. 1 §
Rolfes M. 2
Birkenkamp K. 2
Boulware D. 2
1 Infectious Diseases Institute - Makerere University, Research, Kampala, Uganda
2 Division of Infectious Diseases and International Medicine, University of Minnesota, Department of Medicine, Minneapolis, United States
§ Presenting author email: radha.rajasingham@gmail.com
Electronic publication date: 2012 Oct 22
Publication date: 2012
Volume: 15
Issue: Suppl 3
Electronic Location ID: 18439-14
Copyright: © 2012 R. Rajasingham et al.
Copyright year: 2012
License: This is an open-access article distributed under the terms of the Creative Commons Attribution License, which permits unrestricted use, distribution, and reproduction in any medium, provided the original work is properly cited.
License URL: http://creativecommons.org/licenses/by/2.0/

JOURNAL INFORMATION
==============================
NLM Title Abbreviation: J Int AIDS Soc
Journal ID: J Int AIDS Soc
NLM Journal ID: 101478566
Journal ID: JIAS

Journal of the International AIDS Society

EISSN: 1758-2652
Publisher: Wiley

ARTICLE INFORMATION
==============================
Article ID: 18439-15
Subjects: B12 - Opportunistic infections (excluding tuberculosis)

MOAB0103: Determinants of penicilliosis seasonality in Ho Chi Minh City, Vietnam

Bulterys P. 1 §
Le T. 2 3
Quang V.M. 4
Nelson K. 5
Lloyd-Smith J. 6 7
1 University of California Los Angeles, David Geffen School of Medicine, Medical Scientist Training Program, Los Angeles, United States
2 Oxford University Clinical Research Unit, Wellcome Trust Major Overseas Program, Ho Chi Minh City, Viet Nam
3 University of Hawaii at Manoa, Hawaii Center for AIDS, Honolulu, United States
4 Hospital for Tropical Diseases, Ho Chi Minh City, Viet Nam
5 Johns Hopkins Bloomberg School of Public Health, Department of Epidemiology, Baltimore, United States
6 University of California Los Angeles, Department of Ecology and Evolutionary Biology, Los Angeles, United States
7 National Institutes of Health, Fogarty International Center, Bethesda, United States
§ Presenting author email: pbulterys@mednet.ucla.edu
Electronic publication date: 2012 Oct 22
Publication date: 2012
Volume: 15
Issue: Suppl 3
Electronic Location ID: 18439-15
Copyright: © 2012 P. Bulterys et al.
Copyright year: 2012
License: This is an open-access article distributed under the terms of the Creative Commons Attribution License, which permits unrestricted use, distribution, and reproduction in any medium, provided the original work is properly cited.
License URL: http://creativecommons.org/licenses/by/2.0/

JOURNAL INFORMATION
==============================
NLM Title Abbreviation: J Int AIDS Soc
Journal ID: J Int AIDS Soc
NLM Journal ID: 101478566
Journal ID: JIAS

Journal of the International AIDS Society

EISSN: 1758-2652
Publisher: Wiley

ARTICLE INFORMATION
==============================
Article ID: 18439-16
Subjects: B13 - Tuberculosis (TB)

MOAB0301: Relationship between weight, efavirenz (EFV) concentrations and virologic suppression in HIV+ patients on rifampin (RIF)-based TB treatment in the ACTG 5221 STRIDE study

Luetkemeyer A.F. 1 §
Rosenkranz S.L. 2
Lu D. 2
Lizak P.S. 3
Ive P. 4
Swindells S. 5
Benson C.A. 6
Grinsztejn B. 7
Sanne I.M. 4
Havlir D.V. 1
Aweeka F. 3
Adult AIDS Clinical Trials Group A5221
1 San Francisco General Hospital, University of California at San Francisco, Division of HIV/AIDS, San Francisco, United States
2 Statistical Data Analysis Center, Harvard School of Public Health, Boston, United States
3 San Francisco General Hospital, University of California at San Francisco, Department of Clinical Pharmacy, San Francisco, United States
4 University of Witswatersrand, Clinical HIV Research Unit, Johannesburg, South Africa
5 University of Nebraska at Omaha, Infectious Diseases, Omaha, United States
6 University of California at San Diego (UCSD), Division of Infectious Diseases, San Diego, United States
7 Fundação Oswaldo Cruz, STD/AIDS Clinical Research Laboratory, Rio de Janeiro, Brazil
§ Presenting author email: aluetkemeyer@php.ucsf.edu
Electronic publication date: 2012 Oct 22
Publication date: 2012
Volume: 15
Issue: Suppl 3
Electronic Location ID: 18439-16
Copyright: © 2012 A.F. Luetkemeyer et al.
Copyright year: 2012
License: This is an open-access article distributed under the terms of the Creative Commons Attribution License, which permits unrestricted use, distribution, and reproduction in any medium, provided the original work is properly cited.
License URL: http://creativecommons.org/licenses/by/2.0/

JOURNAL INFORMATION
==============================
NLM Title Abbreviation: J Int AIDS Soc
Journal ID: J Int AIDS Soc
NLM Journal ID: 101478566
Journal ID: JIAS

Journal of the International AIDS Society

EISSN: 1758-2652
Publisher: Wiley

ARTICLE INFORMATION
==============================
Article ID: 18439-17
Subjects: B13 - Tuberculosis (TB)

MOAB0302: Tolerability among HIV-positive persons of three months of once-weekly rifapentine+INH (3HP) versus 9 months of daily INH (9H) for treatment of latent tuberculosis infection: the PREVENT TB Study (TBTC Study 26/ACTG 5259)

Sterling T. 1 §
Benson C. 2
Shang N. 3
Miro J. 4
Grinsztejn B. 5
Chaisson R. 6
Lucchetti A. 7
Sanchez J. 8
Scott N. 3
Villarino E. 3
AIDS Clinical Trials Group, Tuberculosis Trials Consortium
1 Vanderbilt University, Nashville, United States
2 University of California-San Diego (UCSD), San Diego, United States
3 Centers for Disease Control & Prevention, Atlanta, United States
4 Agencia de Salut Publica, Barcelona, Spain
5 Fundaao Oswaldo Cruz, IPEC Evandro Chagas, Rio de Janeiro, Brazil
6 Johns Hopkins University, Baltimore, United States
7 Inmensa, Lima, Peru
8 Impacta, Lima, Peru
§ Presenting author email: timothy.sterling@vanderbilt.edu
Electronic publication date: 2012 Oct 22
Publication date: 2012
Volume: 15
Issue: Suppl 3
Electronic Location ID: 18439-17
Copyright: © 2012 T. Sterling et al.
Copyright year: 2012
License: This is an open-access article distributed under the terms of the Creative Commons Attribution License, which permits unrestricted use, distribution, and reproduction in any medium, provided the original work is properly cited.
License URL: http://creativecommons.org/licenses/by/2.0/

JOURNAL INFORMATION
==============================
NLM Title Abbreviation: J Int AIDS Soc
Journal ID: J Int AIDS Soc
NLM Journal ID: 101478566
Journal ID: JIAS

Journal of the International AIDS Society

EISSN: 1758-2652
Publisher: Wiley

ARTICLE INFORMATION
==============================
Article ID: 18439-18
Subjects: B13 - Tuberculosis (TB)

MOAB0303: Efavirenz (EFV) concentrations in pregnant women taking EFV-based antiretroviral therapy (ART) with and without rifampin-containing tuberculosis (TB) treatment: the TSHEPISO Study Team

McIlleron H. 1
Martinson N. 2 3
Denti P. 1
Mashabela F. 2
Hunt J. 3
Shembe S. 2
Hull J. 4
Haas D.W. 5
Msandiwa R. 2
Cohn S. 3
Chaisson R. 3
Dooley K.E. 3 §
TSHEPISO Study Team
1 University of Cape Town, Cape Town, South Africa
2 Perinatal HIV Research Unit (PHRU), University of the Witwatersrand, Soweto, South Africa
3 Johns Hopkins University School of Medicine, Baltimore, United States
4 Chris Hani Baragwanath Hospital and University of the Witwatersrand, Department of Obstetrics, Soweto, South Africa
5 Vanderbilt University, Nashville, United States
§ Presenting author email: kdooley1@jhmi.edu
Electronic publication date: 2012 Oct 22
Publication date: 2012
Volume: 15
Issue: Suppl 3
Electronic Location ID: 18439-18
Copyright: © 2012 K.E. Dooley et al.
Copyright year: 2012
License: This is an open-access article distributed under the terms of the Creative Commons Attribution License, which permits unrestricted use, distribution, and reproduction in any medium, provided the original work is properly cited.
License URL: http://creativecommons.org/licenses/by/2.0/

JOURNAL INFORMATION
==============================
NLM Title Abbreviation: J Int AIDS Soc
Journal ID: J Int AIDS Soc
NLM Journal ID: 101478566
Journal ID: JIAS

Journal of the International AIDS Society

EISSN: 1758-2652
Publisher: Wiley

ARTICLE INFORMATION
==============================
Article ID: 18439-19
Subjects: B13 - Tuberculosis (TB)

MOAB0304: Pharmacokinetic interaction between the investigational anti-tuberculosis agent TMC207 and rifampicin or rifapentin

Everitt D. 1 §
Winter H. 2
Egizi E. 1
1 TB Alliance, New York, United States
2 University of Otago, Dunedin, New Zealand
§ Presenting author email: dan.everitt@tballiance.org
Electronic publication date: 2012 Oct 22
Publication date: 2012
Volume: 15
Issue: Suppl 3
Electronic Location ID: 18439-19
Copyright: © 2012 D. Everitt et al.
Copyright year: 2012
License: This is an open-access article distributed under the terms of the Creative Commons Attribution License, which permits unrestricted use, distribution, and reproduction in any medium, provided the original work is properly cited.
License URL: http://creativecommons.org/licenses/by/2.0/

JOURNAL INFORMATION
==============================
NLM Title Abbreviation: J Int AIDS Soc
Journal ID: J Int AIDS Soc
NLM Journal ID: 101478566
Journal ID: JIAS

Journal of the International AIDS Society

EISSN: 1758-2652
Publisher: Wiley

ARTICLE INFORMATION
==============================
Article ID: 18439-20
Subjects: B13 - Tuberculosis (TB)

MOAB0305: A phase 2 trial of novel anti-tuberculosis regimens with increased efficacy and low potential to interact adversely with antiretroviral therapy

Everitt D. 1
Murray S. 2 §
Diacon A. 3
Dawson R. 4
Hutchings J. 4
Van Niekerk C. 5
Egizi E. 2
Becker P. 6
1 TB Alliance, Research & Development, New York, United States
2 TB Alliance, New York, United States
3 Stellenbosch University, Cape Town, South Africa
4 University of Cape Town, Cape Town, South Africa
5 TB Alliance, Pretoria, South Africa
6 Medical Research Council of South Africa, Johannesburg, South Africa
§ Presenting author email: stephen.murray@tballiance.org
Electronic publication date: 2012 Oct 22
Publication date: 2012
Volume: 15
Issue: Suppl 3
Electronic Location ID: 18439-20
Copyright: © 2012 S. Murray et al.
Copyright year: 2012
License: This is an open-access article distributed under the terms of the Creative Commons Attribution License, which permits unrestricted use, distribution, and reproduction in any medium, provided the original work is properly cited.
License URL: http://creativecommons.org/licenses/by/2.0/

JOURNAL INFORMATION
==============================
NLM Title Abbreviation: J Int AIDS Soc
Journal ID: J Int AIDS Soc
NLM Journal ID: 101478566
Journal ID: JIAS

Journal of the International AIDS Society

EISSN: 1758-2652
Publisher: Wiley

ARTICLE INFORMATION
==============================
Article ID: 18439-21
Subjects: B13 - Tuberculosis (TB)

THAB0102: Impact of HIV on early MDR-TB treatment outcomes in Botswana

Hafkin J. 1 §
Modongo C. 2
Newcomb C. 1
Lowenthal E. 3
MacGregor R.R. 1
Steenhoff A. 2 3
Friedman H. 1
Bisson G. 1
1 University of Pennsylvania, Philadelphia, United States
2 Botswana UPenn Partnership, Gaborone, Botswana
3 Children's Hospital of Philadelphia, Philadelphia, United States
§ Presenting author email: hafkin@gmail.com
Electronic publication date: 2012 Oct 22
Publication date: 2012
Volume: 15
Issue: Suppl 3
Electronic Location ID: 18439-21
Copyright: © 2012 J. Hafkin et al.
Copyright year: 2012
License: This is an open-access article distributed under the terms of the Creative Commons Attribution License, which permits unrestricted use, distribution, and reproduction in any medium, provided the original work is properly cited.
License URL: http://creativecommons.org/licenses/by/2.0/

JOURNAL INFORMATION
==============================
NLM Title Abbreviation: J Int AIDS Soc
Journal ID: J Int AIDS Soc
NLM Journal ID: 101478566
Journal ID: JIAS

Journal of the International AIDS Society

EISSN: 1758-2652
Publisher: Wiley

ARTICLE INFORMATION
==============================
Article ID: 18439-22
Subjects: B13 - Tuberculosis (TB)

THAB0103: High cost of Xpert MTB/RIF testing per excess tuberculosis case diagnosed at Partners in Hope Medical Center, a public-private HIV care clinic in Lilongwe, Malawi. Comparison with fluorescence microscopy in a well-equipped and experienced real world AFB laboratory

Fitzgerald D. 1 2
Jansen P. 1
Chipungu C. 1
Dindi V. 1
Fielder J. 1
Pfaff C. 1 2 §
1 Partners in Hope, Lilongwe, Malawi
2 University of California, Los Angeles, Program in Global Health, Los Angeles, United States
§ Presenting author email: colinpfaff@yahoo.co.uk
Electronic publication date: 2012 Oct 22
Publication date: 2012
Volume: 15
Issue: Suppl 3
Electronic Location ID: 18439-22
Copyright: © 2012 C. Pfaff et al.
Copyright year: 2012
License: This is an open-access article distributed under the terms of the Creative Commons Attribution License, which permits unrestricted use, distribution, and reproduction in any medium, provided the original work is properly cited.
License URL: http://creativecommons.org/licenses/by/2.0/

JOURNAL INFORMATION
==============================
NLM Title Abbreviation: J Int AIDS Soc
Journal ID: J Int AIDS Soc
NLM Journal ID: 101478566
Journal ID: JIAS

Journal of the International AIDS Society

EISSN: 1758-2652
Publisher: Wiley

ARTICLE INFORMATION
==============================
Article ID: 18439-23
Subjects: B13 - Tuberculosis (TB)

THAB0105: Implementing Xpert® MTB/RIF in rural Zimbabwe: impact on diagnosis of smear-negative TB and time-to-initiation of TB treatment in smear-negative patients co-infected with HIV

Bygrave H. 1
Simons S. 2
Munyaradzi D. 2
Nyagadza B. 2
Metcalf C. 1
Ncube K. 3
Van Den Broucke S. 2 §
1 Medecins Sans Frontieres, South African Medical Unit, Cape Town, South Africa
2 Medecins Sans Frontieres, Harare, Zimbabwe
3 Ministry of Health and Child Welfare Zimbabwe, Buhera, Zimbabwe
§ Presenting author email: helen.bygrave@joburg.msf.org
Electronic publication date: 2012 Oct 22
Publication date: 2012
Volume: 15
Issue: Suppl 3
Electronic Location ID: 18439-23
Copyright: © 2012 S. Van Den Broucke et al.
Copyright year: 2012
License: This is an open-access article distributed under the terms of the Creative Commons Attribution License, which permits unrestricted use, distribution, and reproduction in any medium, provided the original work is properly cited.
License URL: http://creativecommons.org/licenses/by/2.0/

JOURNAL INFORMATION
==============================
NLM Title Abbreviation: J Int AIDS Soc
Journal ID: J Int AIDS Soc
NLM Journal ID: 101478566
Journal ID: JIAS

Journal of the International AIDS Society

EISSN: 1758-2652
Publisher: Wiley

ARTICLE INFORMATION
==============================
Article ID: 18439-24
Subjects: B13 - Tuberculosis (TB)

THAB0104: The value of universal TB screening with GeneXpert MTB/RIF in pre-ART patients in Harare

Mupfumi L. 1 2 §
Mason P. 1
Zinyowera S. 3 4
Mutetwa R. 1
1 Biomedical Research & Training Institute, Harare, Zimbabwe
2 University of Zimbabwe College of Health Sciences, Medical Laboratory Sciences, Harare, Zimbabwe
3 National Microbiology Reference Laboratory, Harare, Zimbabwe
4 University of Zimbabwe College of Health Sciences, Medical Microbiology, Harare, Zimbabwe
§ Presenting author email: lmupfumi@gmail.com
Electronic publication date: 2012 Oct 22
Publication date: 2012
Volume: 15
Issue: Suppl 3
Electronic Location ID: 18439-24
Copyright: © 2012 L. Mupfumi et al.
Copyright year: 2012
License: This is an open-access article distributed under the terms of the Creative Commons Attribution License, which permits unrestricted use, distribution, and reproduction in any medium, provided the original work is properly cited.
License URL: http://creativecommons.org/licenses/by/2.0/

JOURNAL INFORMATION
==============================
NLM Title Abbreviation: J Int AIDS Soc
Journal ID: J Int AIDS Soc
NLM Journal ID: 101478566
Journal ID: JIAS

Journal of the International AIDS Society

EISSN: 1758-2652
Publisher: Wiley

ARTICLE INFORMATION
==============================
Article ID: 18439-25
Subjects: B13 - Tuberculosis (TB)

THLBB02: Safety, tolerability and early bactericidal activity in sputum of PNU-100480 (sutezolid) in patients with pulmonary tuberculosis

Wallis R.S. 1 §
Diacon A.H. 2
Dawson R. 3
Venter A. 2
Friedrich S.O. 2
Paige D. 1
Zhu T. 1
Silvia A. 1
Gobey J. 1
Ellery C. 1
Zhang Y. 1
Kadyszewski E. 1
1 Pfizer, Groton, United States
2 Stellenbosch University, Stellenbosch, South Africa
3 U Cape Town, Cape Town, South Africa
§ Presenting author email: robert.wallis@pfizer.com
Electronic publication date: 2012 Oct 22
Publication date: 2012
Volume: 15
Issue: Suppl 3
Electronic Location ID: 18439-25
Copyright: © 2012 R.S. Wallis et al.
Copyright year: 2012
License: This is an open-access article distributed under the terms of the Creative Commons Attribution License, which permits unrestricted use, distribution, and reproduction in any medium, provided the original work is properly cited.
License URL: http://creativecommons.org/licenses/by/2.0/

JOURNAL INFORMATION
==============================
NLM Title Abbreviation: J Int AIDS Soc
Journal ID: J Int AIDS Soc
NLM Journal ID: 101478566
Journal ID: JIAS

Journal of the International AIDS Society

EISSN: 1758-2652
Publisher: Wiley

ARTICLE INFORMATION
==============================
Article ID: 18439-26
Subjects: B13 - Tuberculosis (TB)

THPDB0106: Adverse events in an integrated, home-based treatment program for MDR-TB and HIV in Tugela Ferry, South Africa

Brust J.C.M. 1 §
Shah N.S. 1
van der Merwe T.L. 2 3
Bamber S. 3
Mngadi A. 4
Ning Y. 5
Heo M. 5
Moll A.P. 2 3
Loveday M. 6 7
Lalloo U.G. 7
Friedland G.H. 8
Gandhi N.R. 1
1 Montefiore Medical Center & Albert Einstein College of Medicine, Medicine, Bronx, United States
2 Church of Scotland Hospital, Tugela Ferry, South Africa
3 Philanjalo, Tugela Ferry, South Africa
4 Greytown Hospital, Greytown, South Africa
5 Montefiore Medical Center & Albert Einstein College of Medicine, Bronx, United States
6 South African Medical Research Council, Health Systems Research Unit, Cape Town, South Africa
7 Nelson R Mandela School of Medicine, University of KwaZulu Natal, Durban, South Africa
8 YaleUniversity School of Medicine, New Haven, South Africa
§ Presenting author email: jcmbrust@gmail.com
Electronic publication date: 2012 Oct 22
Publication date: 2012
Volume: 15
Issue: Suppl 3
Electronic Location ID: 18439-26
Copyright: © 2012 J.C.M. Brust et al.
Copyright year: 2012
License: This is an open-access article distributed under the terms of the Creative Commons Attribution License, which permits unrestricted use, distribution, and reproduction in any medium, provided the original work is properly cited.
License URL: http://creativecommons.org/licenses/by/2.0/

JOURNAL INFORMATION
==============================
NLM Title Abbreviation: J Int AIDS Soc
Journal ID: J Int AIDS Soc
NLM Journal ID: 101478566
Journal ID: JIAS

Journal of the International AIDS Society

EISSN: 1758-2652
Publisher: Wiley

ARTICLE INFORMATION
==============================
Article ID: 18439-27
Subjects: B15 - Hepatitis B and D, including treatment

MOAB0101: Prevalence of hepatitis B co-infection and response to antiretroviral therapy among HIV-positive patients in urban Tanzania

Hawkins C. 1 2
Christian B. 2
Ye J. 3
Nagu T. 4
Aris E. 2 4
Chalamilla G. 2 3
Spiegelman D. 3
Mugusi F. 4
Mehta S. 5
Fawzi W. 2 3
1 Northwestern University, Infectious Diseases, Chicago, United States
2 Management and Development for Health, Dar es Salaam, United Republic of Tanzania
3 Harvard School of Public Health, Boston, United States
4 Muhimbili University of Health and Allied Sciences, Dar es Salaam, United Republic of Tanzania
5 Cornell University, New York, United States
Electronic publication date: 2012 Oct 22
Publication date: 2012
Volume: 15
Issue: Suppl 3
Electronic Location ID: 18439-27
Copyright: © 2012 C. Hawkins et al.
Copyright year: 2012
License: This is an open-access article distributed under the terms of the Creative Commons Attribution License, which permits unrestricted use, distribution, and reproduction in any medium, provided the original work is properly cited.
License URL: http://creativecommons.org/licenses/by/2.0/

JOURNAL INFORMATION
==============================
NLM Title Abbreviation: J Int AIDS Soc
Journal ID: J Int AIDS Soc
NLM Journal ID: 101478566
Journal ID: JIAS

Journal of the International AIDS Society

EISSN: 1758-2652
Publisher: Wiley

ARTICLE INFORMATION
==============================
Article ID: 18439-28
Subjects: B16 - Hepatitis C, including treatment

WEAB0102: Increased risk of hepatic decompensation and hepatocellular carcinoma in HIV/HCV-co-infected patients compared to HCV-mono-infected patients despite combination antiretroviral therapy

Lo Re V. 1 2 §
Tate J. 3 4
Kallan M. 1
Lim J. 3 4
Goetz M. 5
Klein M. 6
Rimland D. 7
Rodriguez-Barradas M. 8
Butt A. 9
Gibert C. 10
Brown S. 11
Kostman J. 1
Strom B. 1
Reddy R. 1
Justice A. 3 4
Localio R. 1
1 Perelman School of Medicine, University of Pennsylvania, Philadelphia, United States
2 Philadelphia VA Medical Center, Philadelphia, United States
3 VA Connecticut Healthcare System, West Haven, United States
4 Yale University School of Medicine, New Haven, United States
5 VA Greater Los Angeles Healthcare System, Los Angeles, United States
6 McGill University Health Centre, Montreal, Canada
7 Atlanta VA Medical Center, Atlanta, United States
8 Michael E. DeBakey VA Medical Center, Houston, United States
9 VA Pittsburgh Healthcare System, Pittsburgh, United States
10 Washington DC VA Medical Center, Washington, United States
11 James J. Peters VA Medical Center, New York, United States
§ Presenting author email: vincentl@mail.med.upenn.edu
Electronic publication date: 2012 Oct 22
Publication date: 2012
Volume: 15
Issue: Suppl 3
Electronic Location ID: 18439-28
Copyright: © 2012 V. Lo Re et al.
Copyright year: 2012
License: This is an open-access article distributed under the terms of the Creative Commons Attribution License, which permits unrestricted use, distribution, and reproduction in any medium, provided the original work is properly cited.
License URL: http://creativecommons.org/licenses/by/2.0/

JOURNAL INFORMATION
==============================
NLM Title Abbreviation: J Int AIDS Soc
Journal ID: J Int AIDS Soc
NLM Journal ID: 101478566
Journal ID: JIAS

Journal of the International AIDS Society

EISSN: 1758-2652
Publisher: Wiley

ARTICLE INFORMATION
==============================
Article ID: 18439-29
Subjects: B16 - Hepatitis C, including treatment

WEAB0103: The addition of nitazoxanide to peginterferon alfa-2a and ribavirin does not significantly improve sustained virologic response in HCV treatment-naïve genotype 1 HIV-1/HCV co-infected subjects: results of ACTG 5269

Amorosa V. 1 2
Umbleja T. 3
Johnson V. 4 5
Kang M. 3
Luetkemeyer A. 6 §
Bardin M. 7
Haas D. 8
Chung R. 9
Yesmin S. 10
Coughlin K. 11
Martinez A. 12
Adams M.B. 13
Alston-Smith B. 12
Tebas P. 2
Peters M. 6
1 Philadelphia Veterans Affairs Medical Center, Medicine, Philadelphia, United States
2 University of Pennsylvania, Medicine, Philadelphia, United States
3 Harvard School of Public Health, Boston, United States
4 Birmingham VA Medical Center, Birmingham, United States
5 University of Alabama Birmingham, Medicine, Birmingham, United States
6 University of San Francisco, San Francisco, United States
7 Romark, Tampa, United States
8 Vanderbilt University, Nashvile, United States
9 Harvard Medical School, Boston, United States
10 ACTG Operations Center, Bethesda, United States
11 Frontiers Science & Technology Research Foundation, Amherst, United States
12 DAIDS, NIAID, NIH, Bethesda, United States
13 University of Rochester, Rochester, United States
§ Presenting author email: marion.peters@ucsf.edu
Electronic publication date: 2012 Oct 22
Publication date: 2012
Volume: 15
Issue: Suppl 3
Electronic Location ID: 18439-29
Copyright: © 2012 A. Luetkemeyer et al.
Copyright year: 2012
License: This is an open-access article distributed under the terms of the Creative Commons Attribution License, which permits unrestricted use, distribution, and reproduction in any medium, provided the original work is properly cited.
License URL: http://creativecommons.org/licenses/by/2.0/

JOURNAL INFORMATION
==============================
NLM Title Abbreviation: J Int AIDS Soc
Journal ID: J Int AIDS Soc
NLM Journal ID: 101478566
Journal ID: JIAS

Journal of the International AIDS Society

EISSN: 1758-2652
Publisher: Wiley

ARTICLE INFORMATION
==============================
Article ID: 18439-30
Subjects: B18 - Prophylaxis of HIV associated infections; vaccines e.g., pneumococcal, hepatitis and HPV, co-trimoxazole prophylaxis and Isoniazid Preventive Therapy (IPT)

WEAB0202: Immunogenicity of the HPV-6, -11, -16, -18 vaccine in HIV-positive young women

Kahn J. 1 §
Xu J. 2
Kapogiannis B. 3
Rudy B. 4
Liu N. 2
Gonin R. 2
Wilson C. 5
Worrell C. 3
Squires K. 6
1 Cincinnati Children's Hospital Medical Center, Pediatrics, Cincinnati, United States
2 Westat Inc., Rockville, United States
3 NICHD/PAMAB, Rockville, United States
4 New York University, New York, United States
5 University of Alabama at Birmingham - School of Public Health, Birmingham, United States
6 Jefferson Medical College, Philadelphia, United States
§ Presenting author email: jessica.kahn@cchmc.org
Electronic publication date: 2012 Oct 22
Publication date: 2012
Volume: 15
Issue: Suppl 3
Electronic Location ID: 18439-30
Copyright: © 2012 J. Kahn et al.
Copyright year: 2012
License: This is an open-access article distributed under the terms of the Creative Commons Attribution License, which permits unrestricted use, distribution, and reproduction in any medium, provided the original work is properly cited.
License URL: http://creativecommons.org/licenses/by/2.0/

JOURNAL INFORMATION
==============================
NLM Title Abbreviation: J Int AIDS Soc
Journal ID: J Int AIDS Soc
NLM Journal ID: 101478566
Journal ID: JIAS

Journal of the International AIDS Society

EISSN: 1758-2652
Publisher: Wiley

ARTICLE INFORMATION
==============================
Article ID: 18439-31
Subjects: B18 - Prophylaxis of HIV associated infections; vaccines e.g., pneumococcal, hepatitis and HPV, co-trimoxazole prophylaxis and Isoniazid Preventive Therapy (IPT)

WEAB0203: Safety and immunogenicity of the quadrivalent human papillomavirus vaccine in HIV-positive women

Kojic E.M. 1
Cespedes M. 2 §
Umbleja T. 3
Kang M. 3
Aberg J. 2
Allen R. 4
Grinsztein B. 5
Firnhaber C. 6
Webster-Cyriaque J. 7
Palefsky J.M. 8
Godfrey C. 9
Saah A.J. 10
Cu-Uvin S. 1
1 Brown University-The Miriam Hospital, Infectious Diseases, Providence, United States
2 New York School of Medicine, New York, United States
3 Harvard School of Public Health, Boston, United States
4 ACTG Operations Center, Silver Spring, United States
5 Chagas Fiocruz, Rio de Janeiro, Brazil
6 University of Witwatersrand, Johannesburg, South Africa
7 University of North Carolina, Chapel Hill, United States
8 University of California at San Francisco, San Francisco, United States
9 NIAID NIH, Bethesda, United States
10 Merck Research Labs, North Wales, United States
§ Presenting author email: ekojic@lifespan.org
Electronic publication date: 2012 Oct 22
Publication date: 2012
Volume: 15
Issue: Suppl 3
Electronic Location ID: 18439-31
Copyright: © 2012 M. Cespedes et al.
Copyright year: 2012
License: This is an open-access article distributed under the terms of the Creative Commons Attribution License, which permits unrestricted use, distribution, and reproduction in any medium, provided the original work is properly cited.
License URL: http://creativecommons.org/licenses/by/2.0/

JOURNAL INFORMATION
==============================
NLM Title Abbreviation: J Int AIDS Soc
Journal ID: J Int AIDS Soc
NLM Journal ID: 101478566
Journal ID: JIAS

Journal of the International AIDS Society

EISSN: 1758-2652
Publisher: Wiley

ARTICLE INFORMATION
==============================
Article ID: 18439-32
Subjects: B18 - Prophylaxis of HIV associated infections; vaccines e.g. pneumococcal, hepatitis and HPV, co-trimoxazole prophylaxis and Isoniazid Preventive Therapy (IPT)

THLBB03: Randomized controlled trial of isoniazid preventive therapy in HIV-infected persons on antiretroviral therapy

Rangaka M.X. 1 2 3 §
Boulle A. 1
Wilkinson R.J. 2 4
van Cutsem G. 1 5
Goemaere E. 5
Goliath R. 2
Titus R. 2
Mathee S. 6
Maartens G. 7
1 University of Cape Town, School of Public Health, Centre for Infectious Disease Epidemiology and Research, Cape Town, South Africa
2 University of Cape Town, Institute of Infectious Diseases and Molecular Medicine, Clinical Infectious Diseases Research Initiative, Cape Town, South Africa
3 London School of Hygiene and Tropical Medicine, (LSHTM), London, United Kingdom
4 Imperial College London, Division of Medicine, London, United Kingdom
5 Medecins Sans Frontieres South Africa, Khayelitsha ART Programme, Khayelitsha, Cape Town, South Africa
6 Provincial Government of the Western Cape, Cape Town, South Africa
7 University of Cape Town, Department of Medicine, Pharmacology Unit, Cape Town, South Africa
§ Presenting author email: mxrangaka@yahoo.co.uk
Electronic publication date: 2012 Oct 22
Publication date: 2012
Volume: 15
Issue: Suppl 3
Electronic Location ID: 18439-32
Copyright: © 2012 M.X. Rangaka et al.
Copyright year: 2012
License: This is an open-access article distributed under the terms of the Creative Commons Attribution License, which permits unrestricted use, distribution, and reproduction in any medium, provided the original work is properly cited.
License URL: http://creativecommons.org/licenses/by/2.0/

JOURNAL INFORMATION
==============================
NLM Title Abbreviation: J Int AIDS Soc
Journal ID: J Int AIDS Soc
NLM Journal ID: 101478566
Journal ID: JIAS

Journal of the International AIDS Society

EISSN: 1758-2652
Publisher: Wiley

ARTICLE INFORMATION
==============================
Article ID: 18439-33
Subjects: B19 - HIV-associated neurocognitive disorders (HAND) and other neurologic disorders

MOAB0204: Cognitive, neurological and adaptive behaviour functioning among children with perinatally-acquired HIV infection in India

Shet A. 1 §
Holla S. 1
Raman V. 2
Dinakar C. 1
Ashok M. 2
1 St Johns National Academy of Health Sciences, Paediatrics, Bangalore, India
2 St Johns National Academy of Health Sciences, Psychiatry, Bangalore, India
§ Presenting author email: anitashet@gmail.com
Electronic publication date: 2012 Oct 22
Publication date: 2012
Volume: 15
Issue: Suppl 3
Electronic Location ID: 18439-33
Copyright: © 2012 A. Shet et al.
Copyright year: 2012
License: This is an open-access article distributed under the terms of the Creative Commons Attribution License, which permits unrestricted use, distribution, and reproduction in any medium, provided the original work is properly cited.
License URL: http://creativecommons.org/licenses/by/2.0/

JOURNAL INFORMATION
==============================
NLM Title Abbreviation: J Int AIDS Soc
Journal ID: J Int AIDS Soc
NLM Journal ID: 101478566
Journal ID: JIAS

Journal of the International AIDS Society

EISSN: 1758-2652
Publisher: Wiley

ARTICLE INFORMATION
==============================
Article ID: 18439-34
Subjects: B19 - HIV-associated neurocognitive disorders (HAND) and other neurologic disorders

THAB0203: Type 2 diabetes is associated with lower cognitive performances in a cohort of HIV-positive patients. ANRS CO3 Aquitaine Cohort, Bordeaux, France, 2007–2009

Dufouil C. 1 §
Richert L. 1
Bruyand M. 1
Amieva H. 1
Dauchy F.-A. 2
Dartigues J.-F. 1
Neau D. 2
Dabis F. 3
Morlat P. 2
Bonnet F. 2
Chene G. 1
ANRS CO3 Aquitaine Study Group
1 INSERM Center 897, CIC-EC7, Bordeaux, France
2 Bordeaux Hospital, Bordeaux, France
3 INSERM Center 897, Bordeaux, France
§ Presenting author email: carole.dufouil@isped.u-bordeaux2.fr
Electronic publication date: 2012 Oct 22
Publication date: 2012
Volume: 15
Issue: Suppl 3
Electronic Location ID: 18439-34
Copyright: © 2012 C. Dufouil et al.
Copyright year: 2012
License: This is an open-access article distributed under the terms of the Creative Commons Attribution License, which permits unrestricted use, distribution, and reproduction in any medium, provided the original work is properly cited.
License URL: http://creativecommons.org/licenses/by/2.0/

JOURNAL INFORMATION
==============================
NLM Title Abbreviation: J Int AIDS Soc
Journal ID: J Int AIDS Soc
NLM Journal ID: 101478566
Journal ID: JIAS

Journal of the International AIDS Society

EISSN: 1758-2652
Publisher: Wiley

ARTICLE INFORMATION
==============================
Article ID: 18439-35
Subjects: B19 - HIV-associated neurocognitive disorders (HAND) and other neurologic disorders

THAB0204: Stroke in human immunodeficiency virus (HIV) infected patients

Nigo M. 1 §
Walker A. 2
Lucido D. 3
Shah A. 2
Skliut M. 2
Mildvan D. 4
1 Beth Israel Medical Center, and Albert Einstein College of Medicine, Internal Medicine, New York, United States
2 Beth Israel Medical Center, and Albert Einstein College of Medicine, Neurology, New York, United States
3 Beth Israel Medical Center, and Albert Einstein College of Medicine, New York, United States
4 Beth Israel Medical Center, and Albert Einstein College of Medicine, Infectious Diseases, New York, United States
§ Presenting author email: masa25103@gmail.com
Electronic publication date: 2012 Oct 22
Publication date: 2012
Volume: 15
Issue: Suppl 3
Electronic Location ID: 18439-35
Copyright: © 2012 M. Nigo et al.
Copyright year: 2012
License: This is an open-access article distributed under the terms of the Creative Commons Attribution License, which permits unrestricted use, distribution, and reproduction in any medium, provided the original work is properly cited.
License URL: http://creativecommons.org/licenses/by/2.0/

JOURNAL INFORMATION
==============================
NLM Title Abbreviation: J Int AIDS Soc
Journal ID: J Int AIDS Soc
NLM Journal ID: 101478566
Journal ID: JIAS

Journal of the International AIDS Society

EISSN: 1758-2652
Publisher: Wiley

ARTICLE INFORMATION
==============================
Article ID: 18439-36
Subjects: B22 - AIDS-related Kaposi's sarcoma (KS), lymphoma, cervical and anal carcinoma including Human Herpes Virus 8 infection (HHV8)

WEAB0204: HPV genotype attribution of anal neoplasia in HIV-positive MSM: estimating the preventable fraction and disease misclassification

Sahasrabuddhe V. 1 §
Castle P. 2
Follansbee S. 3
Borgonovo S. 3
LaMere B. 3
Tokugawa D. 3
Darragh T. 4
Boyle S. 5
Sadorra M. 5
Tang S. 5
Wentzensen N. 1
1 National Cancer Institute, Division of Cancer Epidemiology and Genetics, Rockville, United States
2 American Society for Clinical Pathology, Washington, United States
3 Kaiser Permanente Northern California, San Francisco, United States
4 University of California at San Francisco, San Francisco, United States
5 Roche Molecular Systems, Pleasanton, United States
§ Presenting author email: vikrant.sahasrabuddhe@nih.gov
Electronic publication date: 2012 Oct 22
Publication date: 2012
Volume: 15
Issue: Suppl 3
Electronic Location ID: 18439-36
Copyright: © 2012 V. Sahasrabuddhe et al.
Copyright year: 2012
License: This is an open-access article distributed under the terms of the Creative Commons Attribution License, which permits unrestricted use, distribution, and reproduction in any medium, provided the original work is properly cited.
License URL: http://creativecommons.org/licenses/by/2.0/

JOURNAL INFORMATION
==============================
NLM Title Abbreviation: J Int AIDS Soc
Journal ID: J Int AIDS Soc
NLM Journal ID: 101478566
Journal ID: JIAS

Journal of the International AIDS Society

EISSN: 1758-2652
Publisher: Wiley

ARTICLE INFORMATION
==============================
Article ID: 18439-37
Subjects: B23 - Clinical trials - phase I/II

TUAB0102: Once-daily maraviroc in combination with ritonavir-boosted atazanavir in treatment-naïve patients infected with CCR5-tropic HIV-1 (study A4001078): 96-week results

Mills A. 1 §
Mildvan D. 2
Podzamczer D. 3
Fätkenheuer G. 4
Leal M. 5
Than S. 6
Valluri S.R. 7
Craig C. 8
Vourvahis M. 7
Heera J. 6
Valdez H. 7
Brown T. 9
Rinehart A. 10
Portsmouth S. 7
1 Anthony Mills MD Inc., Los Angeles, United States
2 Beth Israel Medical Center Division of Infectious Diseases, New York, United States
3 HIV Unit, Infectious Disease Service, Hospital Universitari de Bellvitge, Barcelona, Spain
4 First Department of Internal Medicine, University Hospital of Cologne, Köln, Germany
5 Infectious Disease Service, Virgen del Rocio University Hospital, Seville, Spain
6 Pfizer Inc., Groton, United States
7 Pfizer Inc., New York, United States
8 Pfizer Inc., Sandwich, United Kingdom
9 Division of Endocrinology and Metabolism, Johns Hopkins University, Baltimore, United States
10 ViiV Healthcare, Research Triangle Park, United States
§ Presenting author email: tmills@tonymillsmd.com
Electronic publication date: 2012 Oct 22
Publication date: 2012
Volume: 15
Issue: Suppl 3
Electronic Location ID: 18439-37
Copyright: © 2012 A. Mills et al.
Copyright year: 2012
License: This is an open-access article distributed under the terms of the Creative Commons Attribution License, which permits unrestricted use, distribution, and reproduction in any medium, provided the original work is properly cited.
License URL: http://creativecommons.org/licenses/by/2.0/

JOURNAL INFORMATION
==============================
NLM Title Abbreviation: J Int AIDS Soc
Journal ID: J Int AIDS Soc
NLM Journal ID: 101478566
Journal ID: JIAS

Journal of the International AIDS Society

EISSN: 1758-2652
Publisher: Wiley

ARTICLE INFORMATION
==============================
Article ID: 18439-38
Subjects: B24 - Clinical trials - phase III/post-licensing

TUAB0103: Cobicistat versus ritonavir as pharmacoenhancers in combination with atazanavir plus tenofovir disoproxil fumarate/emtricitabine: phase 3 randomized, double blind, active-controlled trial, week 48 results

Gallant J. 1
Koenig E. 2
Andrade-Villanueva J. 3
Chetchotisakd P. 4
Dejesus E. 5
Antunes F. 6
Arastéh K. 7
Moyle G. 8
Rizzardini G. 9
Fehr J. 10
Liu Y.-P. 11
Zhong L. 11
Callebaut C. 11
Ramanathan S. 11
Szwarcberg J. 11
Rhee M. 11
Cheng A. 11
1 Johns Hopkins School of Medicine, Baltimore, United States
2 Instituto Dominicano de Estudios Virologicos - IDEV-, Santo Domingo, Dominican Republic
3 Hospital Civil, Guadalajara, Mexico
4 Khon Kaen University, Muang District, Thailand
5 Orlando Immunology Center, Orlando, United States
6 Hospital de Santa Maria, Lisboa, Portugal
7 EPIMED/Vivantes Auguste-Viktoria-Klinikum, Berlin, Germany
8 Chelsea & Westminster Hospital, London, United Kingdom
9 Ospedale Luigi Sacco, Milano, Italy
10 University Hospital Zurich, Zurich, Switzerland
11 Gilead Sciences, Foster City, United States
Electronic publication date: 2012 Oct 22
Publication date: 2012
Volume: 15
Issue: Suppl 3
Electronic Location ID: 18439-38
Copyright: © 2012 J. Gallant et al.
Copyright year: 2012
License: This is an open-access article distributed under the terms of the Creative Commons Attribution License, which permits unrestricted use, distribution, and reproduction in any medium, provided the original work is properly cited.
License URL: http://creativecommons.org/licenses/by/2.0/

JOURNAL INFORMATION
==============================
NLM Title Abbreviation: J Int AIDS Soc
Journal ID: J Int AIDS Soc
NLM Journal ID: 101478566
Journal ID: JIAS

Journal of the International AIDS Society

EISSN: 1758-2652
Publisher: Wiley

ARTICLE INFORMATION
==============================
Article ID: 18439-39
Subjects: B24 - Clinical trials - phase III/post-licensing

TUAB0104: SPIRIT study: switching to emtricitabine/rilpivirine/tenofovir df (FTC/RPV/TDF) single-tablet regimen (STR) from a ritonavir-boosted protease inhibitor and two nucleoside reverse transcriptase inhibitors (NRTIS) maintains HIV suppression and improves serum lipids in HIV-1-positive subjects

Palella F. 1
Tebas P. 2
Gazzard B. 3
Ruane P. 4
Shamblaw D. 5
Flamm J. 6
Fisher M. 7
van Lunzen J. 8
Ebrahimi R. 9
White K. 9
Guyer B. 9
Graham H. 9
Fralich T. 9
1 Northwestern University, Feinberg School of Medicine, Chicago, United States
2 University of Pennsylvania, Division of Infectious Diseases, Clinical Trials Unit, Philadelphia, United States
3 Chelsea and Westminster Hospital Foundation Trust, London, United Kingdom
4 Peter J. Ruane, MD, Inc., Los Angeles, United States
5 La Playa Medical Group and Clinical Research, San Diego, United States
6 Kaiser Permanente, Sacramento, United States
7 Brighton and Sussex University Hospitals, Brighton, United Kingdom
8 University Medical Center Hamburg-Eppendorf, Hamburg, Germany
9 Gilead Sciences, Foster City, United States
Electronic publication date: 2012 Oct 22
Publication date: 2012
Volume: 15
Issue: Suppl 3
Electronic Location ID: 18439-39
Copyright: © 2012 F. Palella et al.
Copyright year: 2012
License: This is an open-access article distributed under the terms of the Creative Commons Attribution License, which permits unrestricted use, distribution, and reproduction in any medium, provided the original work is properly cited.
License URL: http://creativecommons.org/licenses/by/2.0/

JOURNAL INFORMATION
==============================
NLM Title Abbreviation: J Int AIDS Soc
Journal ID: J Int AIDS Soc
NLM Journal ID: 101478566
Journal ID: JIAS

Journal of the International AIDS Society

EISSN: 1758-2652
Publisher: Wiley

ARTICLE INFORMATION
==============================
Article ID: 18439-40
Subjects: B24 - Clinical trials - phase III/post-licensing

TUAB0105: Efficacy and safety results from a randomized, double blind, active controlled trial of elvitegravir (once-daily) versus raltegravir (twice-daily) in treatment-experienced HIV-positive patients: long term 96-week data

Elion R. 1 §
Molina J.-M. 2
Arribas-Lopez J.-R. 3
Cooper D. 4
Maggiolo F. 5
Wilkins E. 6
Conway B. 7
Liu Y.-P. 8
Zhong L. 8
Margot N. 8
Szwarcberg J. 8
Rhee M. 9
Cheng A. 8
1 Whitman-Walker Health, Washington, United States
2 Hopital Saint Louis, Service des Maladies infectieuses, Paris, France
3 Hospital Universitario La Paz, Servicio de Medicina Interna, Unidad VIH, Madrid, Spain
4 Kirby Institute, University of New South Wales, Sydney, Australia
5 Ospedali Riuniti di Bergamo, Bergamo, Italy
6 North Manchester General Hospital, Manchester, United Kingdom
7 Vancouver ID Research & Care Centre, Vancouver, Canada
8 Gilead Sciences, Foster City, United States
9 Gilead Sciences, HIV Clinical Research, Foster City, United States
§ Presenting author email: relion@wwc.org
Electronic publication date: 2012 Oct 22
Publication date: 2012
Volume: 15
Issue: Suppl 3
Electronic Location ID: 18439-40
Copyright: © 2012 R. Elion et al.
Copyright year: 2012
License: This is an open-access article distributed under the terms of the Creative Commons Attribution License, which permits unrestricted use, distribution, and reproduction in any medium, provided the original work is properly cited.
License URL: http://creativecommons.org/licenses/by/2.0/

JOURNAL INFORMATION
==============================
NLM Title Abbreviation: J Int AIDS Soc
Journal ID: J Int AIDS Soc
NLM Journal ID: 101478566
Journal ID: JIAS

Journal of the International AIDS Society

EISSN: 1758-2652
Publisher: Wiley

ARTICLE INFORMATION
==============================
Article ID: 18439-41
Subjects: B24 - Clinical trials - phase III/post-licensing

THLBB04: Once-daily dolutegravir (DTG; S/GSK1349572) is non-inferior to raltegravir (RAL) in antiretroviral-naive adults: 48 week results from SPRING-2 (ING113086)

Raffi F. 1 §
Rachlis A. 2
Stellbrink H.-J. 3
Hardy W.D. 4
Torti C. 5
Orkin C. 6
Bloch M. 7
Podzamczer D. 8
Pokrovsky V. 9
Almond S. 10
Margolis D. 11
Min S. 11
The SPRING-2 Team
1 University of Nantes, Nantes, France
2 Sunnybrook & Women's College Health Sciences Centre, Toronto, Canada
3 IPM Study Center, Hamburg, Germany
4 Cedars-Sinai Medical Center, Los Angeles, United States
5 Azienda Ospedaliera Spedali Civili, Brescia, Italy
6 Royal London Hospital, London, United Kingdom
7 Holdsworth House Medical Practice, Darlinghurst, Australia
8 Hospital Universitari de Bellvitge, Barcelona, Spain
9 Russian Federal Guidance Centre of AIDS, Moscow, Russian Federation
10 GlaxoSmithKline, Mississauga, Canada
11 GlaxoSmithKline, Research Triangle Park, United States
§ Presenting author email: francois.raffi@wanadoo.fr
Electronic publication date: 2012 Oct 22
Publication date: 2012
Volume: 15
Issue: Suppl 3
Electronic Location ID: 18439-41
Copyright: © 2012 F. Raffi et al.
Copyright year: 2012
License: This is an open-access article distributed under the terms of the Creative Commons Attribution License, which permits unrestricted use, distribution, and reproduction in any medium, provided the original work is properly cited.
License URL: http://creativecommons.org/licenses/by/2.0/

JOURNAL INFORMATION
==============================
NLM Title Abbreviation: J Int AIDS Soc
Journal ID: J Int AIDS Soc
NLM Journal ID: 101478566
Journal ID: JIAS

Journal of the International AIDS Society

EISSN: 1758-2652
Publisher: Wiley

ARTICLE INFORMATION
==============================
Article ID: 18439-42
Subjects: B25 - Cohort studies

THPDB0103: Assessing the WHO recommended first-line ART regimens for resource-limited settings: comparing the relative virologic efficacy and resistance patterns of TDF/3TC/NVP to AZT/3TC/NVP in a Rwandan cohort

Karasi J.-C. 1 2 §
Musonera F. 2
Iranyumviye K. 2
Servais J.-Y. 1
Devaux C. 1
Binagwaho A. 3
Arendt V. 1
Shafer R. 4
Zolopa A. 4
Schmit J.-C. 1
1 CRP-Santé, Retrovirology Laboratory, Luxembourg-Ville, Luxembourg
2 Rwanda Biomedical Center, National Reference Laboratory, Kigali, Rwanda
3 Ministry of Health, Kigali, Rwanda
4 Division of Infectious Diseases, Stanford University, San Francisco, United States
§ Presenting author email: karasirw@me.com
Electronic publication date: 2012 Oct 22
Publication date: 2012
Volume: 15
Issue: Suppl 3
Electronic Location ID: 18439-42
Copyright: © 2012 J.-C. Karasi et al.
Copyright year: 2012
License: This is an open-access article distributed under the terms of the Creative Commons Attribution License, which permits unrestricted use, distribution, and reproduction in any medium, provided the original work is properly cited.
License URL: http://creativecommons.org/licenses/by/2.0/

JOURNAL INFORMATION
==============================
NLM Title Abbreviation: J Int AIDS Soc
Journal ID: J Int AIDS Soc
NLM Journal ID: 101478566
Journal ID: JIAS

Journal of the International AIDS Society

EISSN: 1758-2652
Publisher: Wiley

ARTICLE INFORMATION
==============================
Article ID: 18439-43
Subjects: B28 - Antiretroviral drug resistance

TUAB0302: Similar prevalence of baseline HIV-1 minority variants among responders and virologic failures, as well as increased detection of HIV-1 minority variants at treatment failure, in rilpivirine patients from the ECHO and THRIVE Phase III studies (post hoc resistance analysis)

Rimsky L. 1
Van Eygen V. 1
Vingerhoets J. 1
Thys K. 1
Aerssens J. 1
Stevens M. 1
Picchio G. 2
1 Janssen Infectious Diseases BVBA, Beerse, Belgium
2 Janssen Research & Development LLC, Titusville, United States
Electronic publication date: 2012 Oct 22
Publication date: 2012
Volume: 15
Issue: Suppl 3
Electronic Location ID: 18439-43
Copyright: © 2012 L. Rimsky et al.
Copyright year: 2012
License: This is an open-access article distributed under the terms of the Creative Commons Attribution License, which permits unrestricted use, distribution, and reproduction in any medium, provided the original work is properly cited.
License URL: http://creativecommons.org/licenses/by/2.0/

JOURNAL INFORMATION
==============================
NLM Title Abbreviation: J Int AIDS Soc
Journal ID: J Int AIDS Soc
NLM Journal ID: 101478566
Journal ID: JIAS

Journal of the International AIDS Society

EISSN: 1758-2652
Publisher: Wiley

ARTICLE INFORMATION
==============================
Article ID: 18439-44
Subjects: B28 - Antiretroviral drug resistance

TUAB0303: Changing patterns of NRTI and PI resistance mutations between 2006 and 2011 in >1,200 ART-experienced South African patients: association with the introduction of tenofovir (TDF) and abacavir (ABC) and with the cumulative effects of LPV/r therapy

van Zyl G. 1 §
Claassen M. 2
Engelbrecht S. 1
de Oliveira T. 3
Preiser W. 1
Wood N. 4
Travers S. 4
Shafer R. 5
1 National Health Laboratory Service & Stellenbosch University, Pathology (Medical Virology), Cape Town, South Africa
2 National Health Laboratory Service (NHLS), Pathology (Medical Virology), Cape Town, South Africa
3 Africa Centre for Health and Population Studies, University of KwaZulu-Natal, Mtubatuba, South Africa
4 South African National Bioinformatics Institute (SANBI), University of Western Cape, Cape Town, South Africa
5 Stanford University Medical Center, Division of Infectious Diseases, Center for AIDS Research, Stanford, United States
§ Presenting author email: guvz@sun.ac.za
Electronic publication date: 2012 Oct 22
Publication date: 2012
Volume: 15
Issue: Suppl 3
Electronic Location ID: 18439-44
Copyright: © 2012 G. van Zyl et al.
Copyright year: 2012
License: This is an open-access article distributed under the terms of the Creative Commons Attribution License, which permits unrestricted use, distribution, and reproduction in any medium, provided the original work is properly cited.
License URL: http://creativecommons.org/licenses/by/2.0/

JOURNAL INFORMATION
==============================
NLM Title Abbreviation: J Int AIDS Soc
Journal ID: J Int AIDS Soc
NLM Journal ID: 101478566
Journal ID: JIAS

Journal of the International AIDS Society

EISSN: 1758-2652
Publisher: Wiley

ARTICLE INFORMATION
==============================
Article ID: 18439-45
Subjects: B28 - Antiretroviral drug resistance

WEPDB0102: Persistent low-level HIV-1 RNA between 20 and 50 copies/ml in antiretroviral treated patients: associated factors and virological outcome

Charpentier C. 1 §
Landman R. 1
Laouénan C. 1
Joly V. 1
Hamet G. 1
Damond F. 1
Brun-Vézinet F. 1
Mentré F. 1
Descamps D. 1
Yeni P. 1
1 Hopital Bichat - Claude-Bernard, Paris, France
§ Presenting author email: charlotte.charpentier@bch.aphp.fr
Electronic publication date: 2012 Oct 22
Publication date: 2012
Volume: 15
Issue: Suppl 3
Electronic Location ID: 18439-45
Copyright: © 2012 C. Charpentier et al.
Copyright year: 2012
License: This is an open-access article distributed under the terms of the Creative Commons Attribution License, which permits unrestricted use, distribution, and reproduction in any medium, provided the original work is properly cited.
License URL: http://creativecommons.org/licenses/by/2.0/

JOURNAL INFORMATION
==============================
NLM Title Abbreviation: J Int AIDS Soc
Journal ID: J Int AIDS Soc
NLM Journal ID: 101478566
Journal ID: JIAS

Journal of the International AIDS Society

EISSN: 1758-2652
Publisher: Wiley

ARTICLE INFORMATION
==============================
Article ID: 18439-46
Subjects: B29 - Pharmacology, pharmacokinetics and pharmacogenomics, role of therapeutic drug monitoring, drug interactions

THLBB01: A randomized multicentre open-label trial to estimate the efficacy and safety of two doses of raltegravir (RAL) to efavirenz (EFV) for the treatment of HIV-TB co-infected patients: results of the ANRS 12 180 Reflate TB trial

Grinsztejn B. 1
De Castro N. 2 3 §
Arnold V. 4
Veloso V. 1
Morgado M. 5
Pilotto J.H. 6
Brites C. 7
Madruga J.V. 8
Barcellos N. 9
Riegel Santos B. 10
Vorsatz C. 1
Grondin C. 4
Santini-Oliveira M. 1
Patey O. 11
Delaugerre C. 2 3
Chêne G. 4
Molina J.-M. 2 3
ANRS 12 180 Reflate TB study group
1 Instituto de Pesquisa Clinica Evandro Chagas, Fiocruz, Laboratório de Pesquisa Clínica em DST/AIDS, Rio de Janeiro, Brazil
2 University of Paris Diderot, Sorbonne, Paris Cité, INSERM U941, Paris, France
3 Hospital Saint-Louis, AP-HP, Paris, France
4 University Bordeaux, INSERM U897, Bordeaux, France
5 Instituto Oswaldo Cruz-FIOCRUZ, Laboratory of AIDS and Molecular Immunology, Rio de Janeiro, Brazil
6 Hospital Geral de Nova Iguaçu, Departamento de DST/AIDS, Nova Iguaçu, Brazil
7 Hospital Universitário Prof. Edgar Santos, Laboratório de Pesquisa em Doenças Infecciosas, Salvador, Brazil
8 Centro de Referência e Treinamento DST/AIDS, Unidade de Pesquisa, São Paulo, Brazil
9 Health State Secretariat Hospital Sanatório Partenon, Porto Alegre, Brazil
10 Hospital Nossa Senhora da Conceição, Serviço de Infectologia, Porto Alegre, Brazil
11 C.H.G Villeneuve St George, Department of Internal and Tropical Medicine, Villeneuve St George, France
§ Presenting author email: nathalie.de-castro@sls.ap-hop-paris.fr
Electronic publication date: 2012 Oct 22
Publication date: 2012
Volume: 15
Issue: Suppl 3
Electronic Location ID: 18439-46
Copyright: © 2012 N. De Castro et al.
Copyright year: 2012
License: This is an open-access article distributed under the terms of the Creative Commons Attribution License, which permits unrestricted use, distribution, and reproduction in any medium, provided the original work is properly cited.
License URL: http://creativecommons.org/licenses/by/2.0/

JOURNAL INFORMATION
==============================
NLM Title Abbreviation: J Int AIDS Soc
Journal ID: J Int AIDS Soc
NLM Journal ID: 101478566
Journal ID: JIAS

Journal of the International AIDS Society

EISSN: 1758-2652
Publisher: Wiley

ARTICLE INFORMATION
==============================
Article ID: 18439-47
Subjects: B29 - Pharmacology, pharmacokinetics and pharmacogenomics, role of therapeutic drug monitoring, drug interactions

TUPDB0101: Atazanavir pharmacokinetics and efficacy and safety outcomes by sex in AIDS Clinical Trials Group Study 5202 (A5202)

Venuto C. 1 2 §
Mollan K. 3
Ma Q. 2
Daar E. 4
Sax P. 5
Fischl M. 6
Collier A. 7
Smith K. 8
Lu D. 3
Tierney C. 3
Morse G. 2
ACTG 5202 Protocol Team
1 University of Rochester, Center for Human Experimental Therapeutics, Rochester, United States
2 University at Buffalo, Buffalo, United States
3 Harvard School of Public Health, Center for Biostatistics in AIDS Research, Boston, United States
4 Harbor-UCLA Medical Center, Division of HIV Medicine, Los Angeles, United States
5 Brigham and Women's Hospital, Harvard Medical School, Boston, United States
6 University of Miami School of Medicine, Miami, United States
7 University of Washington at Harborview Medical Center, Seattle, United States
8 Rush University Medical Center, Chicago, United States
§ Presenting author email: charles.venuto@chet.rochester.edu
Electronic publication date: 2012 Oct 22
Publication date: 2012
Volume: 15
Issue: Suppl 3
Electronic Location ID: 18439-47
Copyright: © 2012 C. Venuto et al.
Copyright year: 2012
License: This is an open-access article distributed under the terms of the Creative Commons Attribution License, which permits unrestricted use, distribution, and reproduction in any medium, provided the original work is properly cited.
License URL: http://creativecommons.org/licenses/by/2.0/

JOURNAL INFORMATION
==============================
NLM Title Abbreviation: J Int AIDS Soc
Journal ID: J Int AIDS Soc
NLM Journal ID: 101478566
Journal ID: JIAS

Journal of the International AIDS Society

EISSN: 1758-2652
Publisher: Wiley

ARTICLE INFORMATION
==============================
Article ID: 18439-48
Subjects: B29 - Pharmacology, pharmacokinetics and pharmacogenomics, role of therapeutic drug monitoring, drug interactions

TUPDB0102: Pharmacogenetics of intrapartum single-dose nevirapine (SD NVP) in AIDS Clinical Trials Group (ACTG) Protocol A5207

Haas D. 1 §
Acosta E. 2
Vardhanabhuti S. 3
Ribaudo H. 3
Severe P. 4
Lalloo U. 5
Kumarasamy N. 6
Taulo F. 7
Kabanda J. 8
Oneko O. 9
Ive P. 10
Sambarey P. 11
Chan E. 3
Hitti J. 12
McMahon D. 13
for the AIDS Clinical Trials Group
1 Vanderbilt University, Nashville, United States
2 University of Alabama at Birmingham, Birmingham, United States
3 Statistical Data Analysis Center, Harvard School of Public Health, Boston, United States
4 Groupe Haitien d'Etude du Sarcome de Kaposi et des Infections Opportunistes (GHESKIO), Port-au-Prince, Haiti
5 Nelson R. Mandela School of Medicine, University of KwaZulu Natal, Durban, South Africa
6 YRGCARE Medical Centre, VHS, Chennai, India
7 Malawi College of Medicine, Johns Hopkins University Research Project, Blantyre, Malawi
8 Joint Clinical Research Centre, Kampala, Uganda
9 Kilimanjaro Christian Medical Centre, Moshi, United Republic of Tanzania
10 University of Witwatersrand, Helen Joseph Hospital, Johannesburg, South Africa
11 BJ Medical College, Pune, India
12 University of Washington, Seattle, United States
13 University of Pittsburgh, Pittsburgh, United States
§ Presenting author email: david.w.haas@vanderbilt.edu
Electronic publication date: 2012 Oct 22
Publication date: 2012
Volume: 15
Issue: Suppl 3
Electronic Location ID: 18439-48
Copyright: © 2012 D. Haas et al.
Copyright year: 2012
License: This is an open-access article distributed under the terms of the Creative Commons Attribution License, which permits unrestricted use, distribution, and reproduction in any medium, provided the original work is properly cited.
License URL: http://creativecommons.org/licenses/by/2.0/

JOURNAL INFORMATION
==============================
NLM Title Abbreviation: J Int AIDS Soc
Journal ID: J Int AIDS Soc
NLM Journal ID: 101478566
Journal ID: JIAS

Journal of the International AIDS Society

EISSN: 1758-2652
Publisher: Wiley

ARTICLE INFORMATION
==============================
Article ID: 18439-49
Subjects: B29 - Pharmacology, pharmacokinetics and pharmacogenomics, role of therapeutic drug monitoring, drug interactions

TUPDB0104: A single nucleotide polymorphism in CYP2B6 leads to >3-fold increases in efavirenz concentrations in intensive pharmacokinetic curves and hair samples

Gandhi M. 1 §
Greenblatt R. 2
Bacchetti P. 3
Jin C. 3
Cohen M. 4
Dehovitz J. 5
Anastos K. 6
Gange S. 7
Liu C. 8
Hanson S. 9
Aouizerat B. 9
Women's Interagency HIV Study (WIHS)
1 University of California at San Francisco, Medicine, San Francisco, United States
2 University of California at San Francisco, Clinical Pharmacy, San Francisco, United States
3 University of California at San Francisco, Epidemiology and Biostatistics, San Francisco, United States
4 Rush University Medical Center, Medicine, Chicago, United States
5 SUNY Downstate Medical Center, Medicine, Brooklyn, United States
6 Albert Einstein College of Medicine, Medicine, Bronx, United States
7 Johns Hopkins Bloomberg Schoolof Public Health, Baltimore, United States
8 Georgetown University, Medicine, Washington, United States
9 University of California at San Francisco, Nursing, San Francisco, United States
§ Presenting author email: monica.gandhi@ucsf.edu
Electronic publication date: 2012 Oct 22
Publication date: 2012
Volume: 15
Issue: Suppl 3
Electronic Location ID: 18439-49
Copyright: © 2012 M. Gandhi et al.
Copyright year: 2012
License: This is an open-access article distributed under the terms of the Creative Commons Attribution License, which permits unrestricted use, distribution, and reproduction in any medium, provided the original work is properly cited.
License URL: http://creativecommons.org/licenses/by/2.0/

JOURNAL INFORMATION
==============================
NLM Title Abbreviation: J Int AIDS Soc
Journal ID: J Int AIDS Soc
NLM Journal ID: 101478566
Journal ID: JIAS

Journal of the International AIDS Society

EISSN: 1758-2652
Publisher: Wiley

ARTICLE INFORMATION
==============================
Article ID: 18439-50
Subjects: B29 - Pharmacology, pharmacokinetics and pharmacogenomics, role of therapeutic drug monitoring, drug interactions

TUPDB0105: Comparison of the in vivo pharmacokinetics and in vitro dissolution of raltegravir tablets in HIV-positive patients given the drug by swallowing or by chewing

Gervasoni C. 1 §
Baldelli S. 1
Cerea M. 2
Meraviglia P. 1
Landonio S. 1
Simioni M. 1
Gazzaniga A. 2
Galli M. 2
Rizzardini G. 1
Clementi E. 2
Cattaneo D. 1
1 Luigi Sacco Hospital, Milano, Italy
2 Università degli Studi di Milano, Milano, Italy
§ Presenting author email: cristina.gervasoni@unimi.it
Electronic publication date: 2012 Oct 22
Publication date: 2012
Volume: 15
Issue: Suppl 3
Electronic Location ID: 18439-50
Copyright: © 2012 C. Gervasoni et al.
Copyright year: 2012
License: This is an open-access article distributed under the terms of the Creative Commons Attribution License, which permits unrestricted use, distribution, and reproduction in any medium, provided the original work is properly cited.
License URL: http://creativecommons.org/licenses/by/2.0/

JOURNAL INFORMATION
==============================
NLM Title Abbreviation: J Int AIDS Soc
Journal ID: J Int AIDS Soc
NLM Journal ID: 101478566
Journal ID: JIAS

Journal of the International AIDS Society

EISSN: 1758-2652
Publisher: Wiley

ARTICLE INFORMATION
==============================
Article ID: 18439-51
Subjects: B31 - When to start therapy?

THLBB05: Effect of early versus delayed initiation of antiretroviral therapy (ART) on clinical outcomes in the HPTN 052 randomized clinical trial

Grinsztejn B. 1 §
Hosseinipour M. 2
Swindells S. 3
Ribaudo H. 4
Eron J. 5
Chen Y.Q. 6
Wang L. 6
Ou S.-S. 6
Anderson M. 6
McCauley M. 7
Gamble T. 8
Kumarasamy N. 9
Hakim J. 10
Kumwenda J. 11
Pilotto J. 12
Godbole S. 13
Chariyalertsak S. 14
Santos B. 15
Mayer K. 16
Eshleman S. 17
Piwowar-Manning E. 18
Cottle L. 6
Makhema J. 19
Mills L. 20
Panchia R. 21
Sanne I. 22
Elharrar V. 23
Havlir D. 24
Cohen M.S. 5
for the HPTN 052/ACTG Study Team
1 Instituto de Pesquisa Clinica Evandro Chagas, Fiocruz, Rio de Janeiro, Brazil
2 UNC Project Malawi and University of North Carolina at Chapel Hill, Lilongwe, Malawi
3 University of Nebraska Medical Center, Omaha, United States
4 Center for Biostatistics in AIDS Research, Harvard School of Public Health, Boston, United States
5 University of North Carolina School of Medicine, Chapel Hill, United States
6 Vaccine and Infectious Disease Division, Fred Hutchinson Cancer Research Center, Seattle, United States
7 FHI 360, Washington, DC, United States
8 FHI 360, Durham, United States
9 YRG CARE Medical Centre, Chennai, India
10 University of Zimbabwe, Department of Medicine, Harare, Zimbabwe
11 College of Medicine Johns Hopkins Project, Blantyre, Malawi
12 Hospital Geral de Nova Iguau and Laboratorio de AIDS e Imunologia Molecular-IOC, Rio de Janeiro, Brazil
13 National AIDS Research Institute (ICMR), Pune, India
14 Research Institute for Health Sciences, Chiang Mai University, Chiang Mai, Thailand
15 Hospital Nossa Senhora da Conceiao, Porto Alegre, Brazil
16 Fenway Health/Harvard Medical School, Boston, United States
17 Johns Hopkins School of Medicine, Department of Pathology, Baltimore, United States
18 Johns Hopkins University School of Medicine, Department of Pathology, Baltimore, United States
19 Botswana Harvard AIDS Institute, Gaborone, Botswana
20 KEMRI-CDC Research and Public Health Collaboration, CDC Division of HIV/AIDS Prevention, Kisumu, Kenya
21 Perinatal HIV Research Unit (PHRU), University of the Witwatersrand, Johannesburg, South Africa
22 University of Witswatersrand, Department of Medicine, Johannesburg, South Africa
23 National Institute of Allergy and Infectious Diseases, National Institutes of Health, Bethesda, United States
24 University of California - San Francisco, San Francisco, United States
§ Presenting author email: beatriz.grinsztejn@gmail.com
Electronic publication date: 2012 Oct 22
Publication date: 2012
Volume: 15
Issue: Suppl 3
Electronic Location ID: 18439-51
Copyright: © 2012 B. Grinsztejn et al.
Copyright year: 2012
License: This is an open-access article distributed under the terms of the Creative Commons Attribution License, which permits unrestricted use, distribution, and reproduction in any medium, provided the original work is properly cited.
License URL: http://creativecommons.org/licenses/by/2.0/

JOURNAL INFORMATION
==============================
NLM Title Abbreviation: J Int AIDS Soc
Journal ID: J Int AIDS Soc
NLM Journal ID: 101478566
Journal ID: JIAS

Journal of the International AIDS Society

EISSN: 1758-2652
Publisher: Wiley

ARTICLE INFORMATION
==============================
Article ID: 18439-52
Subjects: B32 - First line therapy

THPDB0104: Antiretroviral therapy outcomes measured by virologic failure in Nigeria

Kanki P.J. 1 §
Chang C. 1
Meloni S. 1
Rawizza H. 2
Jolayemi T. 3
Banigbe-Aluko B. 3
Okonkwo P. 3
Harvard/APIN PEPFAR team
1 Harvard School of Public Health, Immunology and Infectious Disease, Boston, United States
2 Brigham & Women's Hospital, Boston, United States
3 AIDS Prevention Initiative In Nigeria, Ltd./Gte., Abuja, Nigeria
§ Presenting author email: pkanki@hsph.harvard.edu
Electronic publication date: 2012 Oct 22
Publication date: 2012
Volume: 15
Issue: Suppl 3
Electronic Location ID: 18439-52
Copyright: © 2012 P.J. Kanki et al.
Copyright year: 2012
License: This is an open-access article distributed under the terms of the Creative Commons Attribution License, which permits unrestricted use, distribution, and reproduction in any medium, provided the original work is properly cited.
License URL: http://creativecommons.org/licenses/by/2.0/

JOURNAL INFORMATION
==============================
NLM Title Abbreviation: J Int AIDS Soc
Journal ID: J Int AIDS Soc
NLM Journal ID: 101478566
Journal ID: JIAS

Journal of the International AIDS Society

EISSN: 1758-2652
Publisher: Wiley

ARTICLE INFORMATION
==============================
Article ID: 18439-53
Subjects: B33 - Second line therapy

THPDB0105: High rates of survival, virologic suppression and immune reconstitution among patients receiving second-line antiretroviral therapy in the Indian national programme

Rewari B.B. 1 §
Shaukat M. 2
Kabra S. 1
Srikantiah P. 3
1 National AIDS and STD Control Organization, New Delhi, India
2 National AIDS Control Organisation, Ministry of HFW, New Delhi, India
3 University of California, HIV/AIDS Division, San Francisco, United States
§ Presenting author email: drbbrewari@yahoo.com
Electronic publication date: 2012 Oct 22
Publication date: 2012
Volume: 15
Issue: Suppl 3
Electronic Location ID: 18439-53
Copyright: © 2012 B.B. Rewari et al.
Copyright year: 2012
License: This is an open-access article distributed under the terms of the Creative Commons Attribution License, which permits unrestricted use, distribution, and reproduction in any medium, provided the original work is properly cited.
License URL: http://creativecommons.org/licenses/by/2.0/

JOURNAL INFORMATION
==============================
NLM Title Abbreviation: J Int AIDS Soc
Journal ID: J Int AIDS Soc
NLM Journal ID: 101478566
Journal ID: JIAS

Journal of the International AIDS Society

EISSN: 1758-2652
Publisher: Wiley

ARTICLE INFORMATION
==============================
Article ID: 18439-54
Subjects: B35 - Management of late presenters

THAB0303: Characteristics of individuals presenting late for care across Europe: results from the Collaboration of Observational HIV Epidemiological Research Europe (COHERE)

Lundgren J. 1 2 §
on behalf of the late presenters working group of the Collaboration of Observational HIV Epidemiological Research in Europe
1 University of Copenhagen, Copenhagen HIV Programme, Copenhagen, Denmark
2 Rigshospitalet, Copenhagen, Denmark
§ Presenting author email: jdl@cphiv.dk
Electronic publication date: 2012 Oct 22
Publication date: 2012
Volume: 15
Issue: Suppl 3
Electronic Location ID: 18439-54
Copyright: © 2012 J. Lundgren et al.
Copyright year: 2012
License: This is an open-access article distributed under the terms of the Creative Commons Attribution License, which permits unrestricted use, distribution, and reproduction in any medium, provided the original work is properly cited.
License URL: http://creativecommons.org/licenses/by/2.0/

JOURNAL INFORMATION
==============================
NLM Title Abbreviation: J Int AIDS Soc
Journal ID: J Int AIDS Soc
NLM Journal ID: 101478566
Journal ID: JIAS

Journal of the International AIDS Society

EISSN: 1758-2652
Publisher: Wiley

ARTICLE INFORMATION
==============================
Article ID: 18439-55
Subjects: B39 - Pharmacokinetics, pharmacodynamics, pharmacogenomics, therapeutic drug monitoring, formulations, drug interactions in children and adolescents

MOAB0202: Lipoprotein lipase genetic variant P- was associated with lower risk of hypertriglyceridemia in children after one year of HAART

Colombero C. 1
Rocco C. 1
Mecikovsky D. 2
Bologna R. 2
Aulicino P. 3
Sen L. 3
Mangano A. 3 §
1 Hospital de Pediatría JP Garrahan, Lab. Biología Celular y Retrovirus, Ciudad de Buenos Aires, Argentina
2 Hospital de Pediatría JP Garrahan, Servicio de Infectología, Ciudad de Buenos Aires, Argentina
3 Hospital de Pediatría JP Garrahan, CONICET, Lab. Biología Celular y Retrovirus, Ciudad de Buenos Aires, Argentina
§ Presenting author email: amangano@garrahan.gov.ar
Electronic publication date: 2012 Oct 22
Publication date: 2012
Volume: 15
Issue: Suppl 3
Electronic Location ID: 18439-55
Copyright: © 2012 A. Mangano et al.
Copyright year: 2012
License: This is an open-access article distributed under the terms of the Creative Commons Attribution License, which permits unrestricted use, distribution, and reproduction in any medium, provided the original work is properly cited.
License URL: http://creativecommons.org/licenses/by/2.0/

JOURNAL INFORMATION
==============================
NLM Title Abbreviation: J Int AIDS Soc
Journal ID: J Int AIDS Soc
NLM Journal ID: 101478566
Journal ID: JIAS

Journal of the International AIDS Society

EISSN: 1758-2652
Publisher: Wiley

ARTICLE INFORMATION
==============================
Article ID: 18439-56
Subjects: B39 - Pharmacokinetics, pharmacodynamics, pharmacogenomics, therapeutic drug monitoring, formulations, drug interactions in children and adolescents

TUAB0201: Tenofovir disoproxil fumarate (TDF) pharmacokinetics (PK) with daily dosing in the first week of life (HPTN 057)

Nielsen-Saines K. 1 §
Mirochnick M. 2
Kumwenda N. 3
Joao E.C. 4
Kreitchmann R. 5
Pinto J. 6
Santos B. 7
Parsons T. 8
Richardson P. 8
Taha T. 3
Mofenson L. 9
Sato P. 10
Kearney B. 11
Fowler M.G. 12
1 David Geffen School of Medicine at UCLA, Los Angeles, United States
2 Boston University, Boston, United States
3 Johns Hopkins Bloomberg School of Public Health, Baltimore, United States
4 Hospital dos Servidores do Estado, Rio de Janeiro, Brazil
5 Irmandade da Santa Casa de Misercordia de Porto Alegre, Porto Alegre, Brazil
6 Universidade Federal de Minas Gerais, Belo Horizonte, Brazil
7 Grupo Hospitalar Conceicao, Porto Alegre, Brazil
8 Johns Hopkins University School of Medicine, Baltimore, United States
9 NICHD/PAMAB, Bethesda, United States
10 NIAID, Bethesda, United States
11 Gilead Sciences, Forest City, United States
12 Johns Hopkins Medical Institutes, Kampala, Uganda
§ Presenting author email: knielsen@mednet.ucla.edu
Electronic publication date: 2012 Oct 22
Publication date: 2012
Volume: 15
Issue: Suppl 3
Electronic Location ID: 18439-56
Copyright: © 2012 K. Nielsen-Saines et al.
Copyright year: 2012
License: This is an open-access article distributed under the terms of the Creative Commons Attribution License, which permits unrestricted use, distribution, and reproduction in any medium, provided the original work is properly cited.
License URL: http://creativecommons.org/licenses/by/2.0/

JOURNAL INFORMATION
==============================
NLM Title Abbreviation: J Int AIDS Soc
Journal ID: J Int AIDS Soc
NLM Journal ID: 101478566
Journal ID: JIAS

Journal of the International AIDS Society

EISSN: 1758-2652
Publisher: Wiley

ARTICLE INFORMATION
==============================
Article ID: 18439-57
Subjects: B39 - Pharmacokinetics, pharmacodynamics, pharmacogenomics, therapeutic drug monitoring, formulations, drug interactions in children and adolescents

TUAB0203: Pharmacokinetics, safety and efficacy of dolutegravir (DTG; S/GSK1349572) in HIV-1-positive adolescents: preliminary analysis from IMPAACT P1093

Hazra R. 1 §
Viani R. 2
Acosta E. 3
Zheng N. 4
Alvero C. 4
O'Gara E. 5
Petzold E. 6
Heckman B. 7
Steimers D. 8
Song I. 8
Piscitelli S. 8
Wiznia A. 9
P1093 Study Team
1 NIH/NICHD, Pediatric Adolescent and Maternal AIDS Branch, Bethesda, United States
2 University of California San Diego School of Medicine, La Jolla, United States
3 University of Alabama at Birmingham School of Medicine, Birmingham, United States
4 Harvard School of Public Health, Boston, United States
5 NIH/NIAID, International Maternal, Adolescent and Pediatric Branch, Bethesda, United States
6 Social & Scientific Systems, Durham, United States
7 Frontier Science and Technology Research Foundation, Inc., Amherst, United States
8 GlaxoSmithKline, Research Triangle Park, United States
9 Jacobi Medical Center, Bronx, United States
§ Presenting author email: hazrar@mail.nih.gov
Electronic publication date: 2012 Oct 22
Publication date: 2012
Volume: 15
Issue: Suppl 3
Electronic Location ID: 18439-57
Copyright: © 2012 R. Hazra et al.
Copyright year: 2012
License: This is an open-access article distributed under the terms of the Creative Commons Attribution License, which permits unrestricted use, distribution, and reproduction in any medium, provided the original work is properly cited.
License URL: http://creativecommons.org/licenses/by/2.0/

JOURNAL INFORMATION
==============================
NLM Title Abbreviation: J Int AIDS Soc
Journal ID: J Int AIDS Soc
NLM Journal ID: 101478566
Journal ID: JIAS

Journal of the International AIDS Society

EISSN: 1758-2652
Publisher: Wiley

ARTICLE INFORMATION
==============================
Article ID: 18439-58
Subjects: B40 - Clinical trials and antiretroviral therapy in children and adolescents

TUAB0202: Pharmacokinetics, safety and antiviral activity of fosamprenavir/ritonavir-containing regimens in HIV-positive four weeks to<two year-old children (48-week data, study APV20002, a prospective, open-label, multi-centre, 48-week cohort study)

Cotton M. 1
Cassim H. 2
Pavía-Ruz N. 3
Ross L. 4
Ford S. 4
Givens N. 5
Cheng K. 5
Sievers J. 5 §
1 Tygerberg Children's Hospital, Tygerberg, South Africa
2 Perinatal HIV Research Unit, Johannesburg, South Africa
3 Universidad Nacional Autonoma de Mexico, Facultad de Medicina, Mexico, DF, Mexico
4 GlaxoSmithKline, Research Triangle Park, United States
5 GlaxoSmithKline, Uxbridge, United Kingdom
§ Presenting author email: jorg.x.sievers@gsk.com
Electronic publication date: 2012 Oct 22
Publication date: 2012
Volume: 15
Issue: Suppl 3
Electronic Location ID: 18439-58
Copyright: © 2012 J. Sievers et al.
Copyright year: 2012
License: This is an open-access article distributed under the terms of the Creative Commons Attribution License, which permits unrestricted use, distribution, and reproduction in any medium, provided the original work is properly cited.
License URL: http://creativecommons.org/licenses/by/2.0/

JOURNAL INFORMATION
==============================
NLM Title Abbreviation: J Int AIDS Soc
Journal ID: J Int AIDS Soc
NLM Journal ID: 101478566
Journal ID: JIAS

Journal of the International AIDS Society

EISSN: 1758-2652
Publisher: Wiley

ARTICLE INFORMATION
==============================
Article ID: 18439-59
Subjects: B40 - Clinical trials and antiretroviral therapy in children and adolescents

TUAB0204: Safety and efficacy of etravirine in HIV-1-infected, treatment-experienced children and adolescents: PIANO 48-week results

Tudor-Williams G. 1
Cahn P. 2
Chokephaibulkit K. 3
Fourie J. 4
Karatzios C. 5
Dincq S. 6
Kakuda T.N. 7
Nijs S. 6
Tambuyzer L. 6
Tomaka F. 7
1 Imperial College Department of Medicine, London, United Kingdom
2 Fundación Huesped, Buenos Aires, Argentina
3 Mahidol University, Bangkok, Thailand
4 Dr Jan Fourie Medical Practice, Dundee, South Africa
5 McGill University Health Centre, Montréal, Canada
6 Janssen Infectious Diseases BVBA, Beerse, Belgium
7 Janssen Research & Development LLC, Titusville, United States
Electronic publication date: 2012 Oct 22
Publication date: 2012
Volume: 15
Issue: Suppl 3
Electronic Location ID: 18439-59
Copyright: © 2012 G. Tudor-Williams et al.
Copyright year: 2012
License: This is an open-access article distributed under the terms of the Creative Commons Attribution License, which permits unrestricted use, distribution, and reproduction in any medium, provided the original work is properly cited.
License URL: http://creativecommons.org/licenses/by/2.0/

JOURNAL INFORMATION
==============================
NLM Title Abbreviation: J Int AIDS Soc
Journal ID: J Int AIDS Soc
NLM Journal ID: 101478566
Journal ID: JIAS

Journal of the International AIDS Society

EISSN: 1758-2652
Publisher: Wiley

ARTICLE INFORMATION
==============================
Article ID: 18439-60
Subjects: B40 - Clinical trials and antiretroviral therapy in children and adolescents

TUAB0205: IMPAACT P1066: raltegravir (RAL) safety and efficacy in HIV infected (+) youth two to 18 years of age through week 48

Nachman S. 1 §
Acosta E. 2
Zheng N. 3
Teppler H. 4
Homony B. 4
Xu X. 4
Alvero C. 3
Handelsman E. 5
Worrell C. 6
Graham B. 7
Toye M. 8
Petzold E. 9
Wiznia A. 10
and the P1066 Group
1 SUNY Stony Brook, Pediatrics, Stony Brook, United States
2 University of Alabama at Birmingham, Birmingham, United States
3 Harvard School of Public Health, Boston, United States
4 Merck, North Wales, United States
5 Division of AIDSNIAID, NIH, Bethesda, United States
6 Natl Inst of Child Hlth and Human Devt, Bethesda, United States
7 Frontier Science Inc, Buffalo, United States
8 Baystate Medical Center, Springfield, United States
9 Social and Scientific Systems, Durham, United States
10 Jacobi Medical Center, Albert Einstein College of Medicine, Bronx, United States
§ Presenting author email: sharon.nachman@stonybrook.edu
Electronic publication date: 2012 Oct 22
Publication date: 2012
Volume: 15
Issue: Suppl 3
Electronic Location ID: 18439-60
Copyright: © 2012 S. Nachman et al.
Copyright year: 2012
License: This is an open-access article distributed under the terms of the Creative Commons Attribution License, which permits unrestricted use, distribution, and reproduction in any medium, provided the original work is properly cited.
License URL: http://creativecommons.org/licenses/by/2.0/

JOURNAL INFORMATION
==============================
NLM Title Abbreviation: J Int AIDS Soc
Journal ID: J Int AIDS Soc
NLM Journal ID: 101478566
Journal ID: JIAS

Journal of the International AIDS Society

EISSN: 1758-2652
Publisher: Wiley

ARTICLE INFORMATION
==============================
Article ID: 18439-61
Subjects: B41 - Adherence in children and adolescents

THAE0101: Success in reaching national pediatric uptake targets and similar adult retention rates in pediatric HIV clinics in the rural Southern Rift Valley (SRV) province of Kenya

Miruka A. 1 §
Achieng R. 1
Aoko A. 1
Tarus J. 1
Sigei C. 1
Yegon P. 1
Maswai J. 1
Sawe F. 1
Shaffer D. 2 3
Crawford K. 3
1 Kenya Medical Research Institute/Walter Reed Project, Kericho, Kenya
2 US Army Medical Research Unit, Kenya (USAMRU-K), Kericho, Kenya
3 US Military HIV Research Program (USMHRP), Bethesda, United States
§ Presenting author email: amiruka@wrp-kch.org
Electronic publication date: 2012 Oct 22
Publication date: 2012
Volume: 15
Issue: Suppl 3
Electronic Location ID: 18439-61
Copyright: © 2012 A. Miruka et al.
Copyright year: 2012
License: This is an open-access article distributed under the terms of the Creative Commons Attribution License, which permits unrestricted use, distribution, and reproduction in any medium, provided the original work is properly cited.
License URL: http://creativecommons.org/licenses/by/2.0/

JOURNAL INFORMATION
==============================
NLM Title Abbreviation: J Int AIDS Soc
Journal ID: J Int AIDS Soc
NLM Journal ID: 101478566
Journal ID: JIAS

Journal of the International AIDS Society

EISSN: 1758-2652
Publisher: Wiley

ARTICLE INFORMATION
==============================
Article ID: 18439-62
Subjects: B42 - Complications of HIV therapy in children and adolescents

MOAB0201: Lipid profile in children randomized to immediate versus deferred nevirapine-based antiretroviral therapy in the PREDICT study

Kanjanavanit S. 1 §
Puthanakit T. 2 3
Kosalaraksa P. 4
Hansudewechakul R. 5
Ngampiyaskul C. 6
Pinyakorn S. 2
Luesomboon W. 7
Vonthanak S. 8
Ananworanich J. 2 9 10
Ruxrungtham K. 2 10
PREDICT study group
1 Nakornping Hospital, Chiang Mai, Thailand
2 HIV Netherlands Australia Thailand (HIV-NAT) Research Collaboration, Thai Red Cross AIDS Research Center, Bangkok, Thailand
3 Department of Pediatrics, Faculty of Medicine, Chulalongkorn University, Bangkok, Thailand
4 Department of Pediatrics, Faculty of Medicine, Khon Kaen University, Khon Kaen, Thailand
5 Chiangrai Prachanukroh Hospital, Chiangrai, Thailand
6 Prapokklao Hospital, Chantaburi, Thailand
7 Queen Savang Vadhana Memorial Hospital, Chonburi, Thailand
8 National Center for HIV/AIDS, Dermatology and STDs, Phnom Penh, Cambodia
9 SEARCH, Thai Red Cross AIDS Research Center, Bangkok, Thailand
10 Department of Medicine, Faculty of Medicine, Chulalongkorn University, Bangkok, Thailand
§ Presenting author email: skanjanavanit@gmail.com
Electronic publication date: 2012 Oct 22
Publication date: 2012
Volume: 15
Issue: Suppl 3
Electronic Location ID: 18439-62
Copyright: © 2012 S. Kanjanavanit et al.
Copyright year: 2012
License: This is an open-access article distributed under the terms of the Creative Commons Attribution License, which permits unrestricted use, distribution, and reproduction in any medium, provided the original work is properly cited.
License URL: http://creativecommons.org/licenses/by/2.0/

JOURNAL INFORMATION
==============================
NLM Title Abbreviation: J Int AIDS Soc
Journal ID: J Int AIDS Soc
NLM Journal ID: 101478566
Journal ID: JIAS

Journal of the International AIDS Society

EISSN: 1758-2652
Publisher: Wiley

ARTICLE INFORMATION
==============================
Article ID: 18439-63
Subjects: B42 - Complications of HIV therapy in children and adolescents

MOAB0203: Mitochondrial function and metabolic abnormalities in children with perinatally-aquired HIV infection in the Pediatric HIV/AIDS Cohort Study (PHACS)

Miller T.L. 1
Wang J. 2
Jacobson D.L. 2
Takemoto J.K. 3
Sharma T. 4
Geffner M.E. 5
Libutti D.E. 3
Siminski S. 6
Dooley L. 6
Somarriba G. 1
Graham P. 1
Gerschenson M. 7 §
1 University of Miami, Pediatrics, Miami, United States
2 Harvard School of Public Health, Boston, United States
3 University of Hawaii Manoa, Honolulu, United States
4 Children's Hospital Boston, Boston, United States
5 Children's Hospital Los Angeles, Los Angeles, United States
6 Frontier Science & Technology Research Foundation, Inc., Amherst, United States
7 Cellular and Molecular Biology, University of Hawaii Manoa, Honolulu, United States
§ Presenting author email: gerschen@hawaii.edu
Electronic publication date: 2012 Oct 22
Publication date: 2012
Volume: 15
Issue: Suppl 3
Electronic Location ID: 18439-63
Copyright: © 2012 M. Gerschenson et al.
Copyright year: 2012
License: This is an open-access article distributed under the terms of the Creative Commons Attribution License, which permits unrestricted use, distribution, and reproduction in any medium, provided the original work is properly cited.
License URL: http://creativecommons.org/licenses/by/2.0/

JOURNAL INFORMATION
==============================
NLM Title Abbreviation: J Int AIDS Soc
Journal ID: J Int AIDS Soc
NLM Journal ID: 101478566
Journal ID: JIAS

Journal of the International AIDS Society

EISSN: 1758-2652
Publisher: Wiley

ARTICLE INFORMATION
==============================
Article ID: 18439-64
Subjects: B42 - Complications of HIV therapy in children and adolescents

MOAB0205: Clinical screening shows high prevalence of peripheral neuropathy in children taking antiretroviral therapy in rural South Africa

van Ramshorst M. 1
Struthers H. 2
McIntyre J.A. 2 3
Peters R.P.H. 1 §
1 Anova Health Institute, Khutso Kurhula Project, Tzaneen, South Africa
2 Anova Health Institute, Johannesburg, South Africa
3 University of Cape Town, School of Public Health, Cape Town, South Africa
§ Presenting author email: petersr@anovahealth.co.za
Electronic publication date: 2012 Oct 22
Publication date: 2012
Volume: 15
Issue: Suppl 3
Electronic Location ID: 18439-64
Copyright: © 2012 R.P.H. Peters et al.
Copyright year: 2012
License: This is an open-access article distributed under the terms of the Creative Commons Attribution License, which permits unrestricted use, distribution, and reproduction in any medium, provided the original work is properly cited.
License URL: http://creativecommons.org/licenses/by/2.0/

JOURNAL INFORMATION
==============================
NLM Title Abbreviation: J Int AIDS Soc
Journal ID: J Int AIDS Soc
NLM Journal ID: 101478566
Journal ID: JIAS

Journal of the International AIDS Society

EISSN: 1758-2652
Publisher: Wiley

ARTICLE INFORMATION
==============================
Article ID: 18439-65
Subjects: B42 - Complications of HIV therapy in children and adolescents

TUPDB0103: Development of the first liquid chromatography-tandem mass spectrometry assay for antiretrovirals in meconium

Himes S. 1 §
Scheidweiler K. 1
Tassiopoulos K. 2
Kacanek D. 3
Hazra R. 4
Rich K. 5
Huestis M. 1
for the Pediatric HIV/AIDS Cohort Study (PHAC)
1 National Institute on Drug Abuse (NIDA), Chemistry and Drug Metabolism, Baltimore, United States
2 Harvard School of Public Health, Epidemiology Department, Boston, United States
3 Harvard School of Public Health, Center for Biostatistics in AIDS Research, Boston, United States
4 National Institute of Child Health and Human Development, Pediatric Adolescent and Maternal AIDS Branch, Bethesda, United States
5 University of Illinois at Chicago, Pediatrics Department, Chicago, United States
§ Presenting author email: sarah.himes@nih.gov
Electronic publication date: 2012 Oct 22
Publication date: 2012
Volume: 15
Issue: Suppl 3
Electronic Location ID: 18439-65
Copyright: © 2012 S. Himes et al.
Copyright year: 2012
License: This is an open-access article distributed under the terms of the Creative Commons Attribution License, which permits unrestricted use, distribution, and reproduction in any medium, provided the original work is properly cited.
License URL: http://creativecommons.org/licenses/by/2.0/

JOURNAL INFORMATION
==============================
NLM Title Abbreviation: J Int AIDS Soc
Journal ID: J Int AIDS Soc
NLM Journal ID: 101478566
Journal ID: JIAS

Journal of the International AIDS Society

EISSN: 1758-2652
Publisher: Wiley

ARTICLE INFORMATION
==============================
Article ID: 18439-66
Subjects: B45 - Cardiovascular disease

THAB0202: Increased platelet activity and immune activation in HIV-positive subjects on antiretroviral therapy is attenuated with low-dose aspirin

O'Brien M. 1
Nardi M.A. 2
Montenont E. 2
Valdes V. 2
Hu L. 2
Merolla M. 2
Gettenberg G. 2
Aberg J. 2
Bhardwaj N. 2
Berger J.S. 2
1 New York University Medical School, Medicine, Infectious Diseases, New York, United States
2 New York University Medical School, New York, United States
Electronic publication date: 2012 Oct 22
Publication date: 2012
Volume: 15
Issue: Suppl 3
Electronic Location ID: 18439-66
Copyright: © 2012 M. O'Brien et al.
Copyright year: 2012
License: This is an open-access article distributed under the terms of the Creative Commons Attribution License, which permits unrestricted use, distribution, and reproduction in any medium, provided the original work is properly cited.
License URL: http://creativecommons.org/licenses/by/2.0/

JOURNAL INFORMATION
==============================
NLM Title Abbreviation: J Int AIDS Soc
Journal ID: J Int AIDS Soc
NLM Journal ID: 101478566
Journal ID: JIAS

Journal of the International AIDS Society

EISSN: 1758-2652
Publisher: Wiley

ARTICLE INFORMATION
==============================
Article ID: 18439-67
Subjects: B48 - Endocrine and metabolic issues (e.g., diabetes, hyperlipidemia)

THAB0201: The interplay of the osteoprotegerin/RANKL axis and dysfunctional HDL in HIV-positive adults: ACTG NWCS 332/A5078 study

Kelesidis T. 1
Kendall M. 2
Yang O. 1
Currier J. 1
1 David Geffen School of Medicine at UCLA, Los Angeles, United States
2 Harvard School of Public Health, Boston, United States
Electronic publication date: 2012 Oct 22
Publication date: 2012
Volume: 15
Issue: Suppl 3
Electronic Location ID: 18439-67
Copyright: © 2012 T. Kelesidis et al.
Copyright year: 2012
License: This is an open-access article distributed under the terms of the Creative Commons Attribution License, which permits unrestricted use, distribution, and reproduction in any medium, provided the original work is properly cited.
License URL: http://creativecommons.org/licenses/by/2.0/

JOURNAL INFORMATION
==============================
NLM Title Abbreviation: J Int AIDS Soc
Journal ID: J Int AIDS Soc
NLM Journal ID: 101478566
Journal ID: JIAS

Journal of the International AIDS Society

EISSN: 1758-2652
Publisher: Wiley

ARTICLE INFORMATION
==============================
Article ID: 18439-68
Subjects: B51 - Immune activation and inflammatory state

THLBB06: Associations of inflammatory markers with AIDS and non-AIDS clinical events after initiation of antiretroviral therapy (ART): AIDS Clinical Trials Group A5224s, a substudy of ACTG A5202

McComsey G.A. 1 §
Kitch D. 2
Sax P.E. 3
Tierney C. 2
Jahed N.C. 4
Melbourne K. 5
Ha B. 6
Brown T.T. 7
Bloom A. 8
Fedarko N. 7
Daar E.S. 9
Adult Aids Clinical Trials Group A5224s
1 Case Western Reserve University, Cleveland, United States
2 Harvard School of Public Health, Boston, United States
3 Brigham and Women's Hospital and Harvard Medical School, Boston, United States
4 Social & Scientific Systems, Inc, Silver Spring, United States
5 Gilead Sciences, Foster City, United States
6 GlaxoSmithKline, Research Triangle, United States
7 Johns Hopkins, Baltimore, United States
8 Frontier Science and Technology Research Foundation, Inc., Amherst, United States
9 Los Angeles Biomedical Research Institute at Harbor-UCLA Medical Center, Torrance, United States
§ Presenting author email: gam9@case.edu
Electronic publication date: 2012 Oct 22
Publication date: 2012
Volume: 15
Issue: Suppl 3
Electronic Location ID: 18439-68
Copyright: © 2012 G.A. McComsey et al.
Copyright year: 2012
License: This is an open-access article distributed under the terms of the Creative Commons Attribution License, which permits unrestricted use, distribution, and reproduction in any medium, provided the original work is properly cited.
License URL: http://creativecommons.org/licenses/by/2.0/

JOURNAL INFORMATION
==============================
NLM Title Abbreviation: J Int AIDS Soc
Journal ID: J Int AIDS Soc
NLM Journal ID: 101478566
Journal ID: JIAS

Journal of the International AIDS Society

EISSN: 1758-2652
Publisher: Wiley

ARTICLE INFORMATION
==============================
Article ID: 18439-69
Subjects: B53 - Ageing in persons with HIV

THAB0205: Comorbidity and ageing in HIV-1 infection: the AGEhIV Cohort Study

Schouten J. 1 2 3 §
Wit F.W. 1 2 4
Stolte I.G. 5
van der Valk M. 4
Geerlings S.E. 4
de Wolf F. 6
Prins M. 5
Reiss P. 1 2 4
on behalf of the Agehiv Cohort Study Group
1 Academic Medical Center (AMC), Global Health, Amsterdam, Netherlands
2 Amsterdam Institute for Global Health and Development (AIGHD), Amsterdam, Netherlands
3 Academic Medical Center (AMC), Neurology, Amsterdam, Netherlands
4 Academic Medical Center (AMC), Infectious Diseases, Amsterdam, Netherlands
5 Public Health Service Amsterdam, Infectious Diseases Research, Amsterdam, Netherlands
6 HIV Monitoring Foundation, Amsterdam, Netherlands
§ Presenting author email: j.schouten@amc.nl
Electronic publication date: 2012 Oct 22
Publication date: 2012
Volume: 15
Issue: Suppl 3
Electronic Location ID: 18439-69
Copyright: © 2012 J. Schouten et al.
Copyright year: 2012
License: This is an open-access article distributed under the terms of the Creative Commons Attribution License, which permits unrestricted use, distribution, and reproduction in any medium, provided the original work is properly cited.
License URL: http://creativecommons.org/licenses/by/2.0/

JOURNAL INFORMATION
==============================
NLM Title Abbreviation: J Int AIDS Soc
Journal ID: J Int AIDS Soc
NLM Journal ID: 101478566
Journal ID: JIAS

Journal of the International AIDS Society

EISSN: 1758-2652
Publisher: Wiley

ARTICLE INFORMATION
==============================
Article ID: 18439-70
Subjects: B57 - Eradication / reservoir depletion

TUAE0104: HIV cure strategies: how good must they be to improve on current antiretroviral therapy?

Sax P. 1 2 §
Sypek A. 3
Morris B. 3
Losina E. 1 2 4
Paltiel A.D. 5
Seage G. 6
Walensky R. 1 2 3
Weinstein M. 6
Eron J. 7 8
Freedberg K. 1 3 6
1 Harvard Medical School, Boston, United States
2 Brigham and Women's Hopsital, Boston, United States
3 Massachusetts General Hospital, Boston, United States
4 Boston University School of Public Health, Boston, United States
5 Yale School of Medicine, New Haven, United States
6 Harvard School of Public Health, Boston, United States
7 Gillings School of Global Public Health, University of North Carolina, Chapel Hill, United States
8 University of North Carolina School of Medicine, Chapel Hill, United States
§ Presenting author email: psax@partners.org
Electronic publication date: 2012 Oct 22
Publication date: 2012
Volume: 15
Issue: Suppl 3
Electronic Location ID: 18439-70
Copyright: © 2012 P. Sax et al.
Copyright year: 2012
License: This is an open-access article distributed under the terms of the Creative Commons Attribution License, which permits unrestricted use, distribution, and reproduction in any medium, provided the original work is properly cited.
License URL: http://creativecommons.org/licenses/by/2.0/

